# Climate-driven environmental changes around 8,200 years ago favoured increases in cetacean strandings and Mediterranean hunter-gatherers exploited them

**DOI:** 10.1038/srep16288

**Published:** 2015-11-17

**Authors:** Marcello A. Mannino, Sahra Talamo, Antonio Tagliacozzo, Ivana Fiore, Olaf Nehlich, Marcello Piperno, Sebastiano Tusa, Carmine Collina, Rosaria Di Salvo, Vittoria Schimmenti, Michael P. Richards

**Affiliations:** 1 Department of Human Evolution, Max Planck Institute for Evolutionary Anthropology, 04103 Leipzig, Germany; 2 Department of Archaeology, School of Culture and Society, Aarhus University, 8270 Højbjerg, Denmark; 3 Soprintendenza al Museo Nazionale Preistorico ed Etnografico ‘Luigi Pigorini’, 00144 Roma, Italy; 4 Department of Anthropology, University of British Columbia, Vancouver, BC V6T1Z1, Canada; 5 Dipartimento di Scienze Storiche, Archeologiche ed Antropologiche dell’Antichità, Sezione di Paletnologia, Università di Roma ‘La Sapienza’, 00185 Roma, Italy; 6 Museo Civico Archeologico ‘Biagio Greco’, 81034 Mondragone (Caserta), Italy; 7 Soprintendenza del Mare, Regione Siciliana, 90133 Palermo, Italy; 8 Università degli Studi ‘Suor Orsola Benincasa’, 80132 Napoli, Italy; 9 Museo Archeologico Regionale ‘Antonino Salinas’, 90133 Palermo, Italy

## Abstract

Cetacean mass strandings occur regularly worldwide, yet the compounded effects of natural and anthropogenic factors often complicate our understanding of these phenomena. Evidence of past stranding episodes may, thus, be essential to establish the potential influence of climate change. Investigations on bones from the site of Grotta dell’Uzzo in North West Sicily (Italy) show that the rapid climate change around 8,200 years ago coincided with increased strandings in the Mediterranean Sea. Stable isotope analyses on collagen from a large sample of remains recovered at this cave indicate that Mesolithic hunter-gatherers relied little on marine resources. A human and a red fox dating to the 8.2-kyr-BP climatic event, however, acquired at least one third of their protein from cetaceans. Numerous carcasses should have been available annually, for at least a decade, to obtain these proportions of meat. Our findings imply that climate-driven environmental changes, caused by global warming, may represent a serious threat to cetaceans in the near future.

The consequences of anthropogenic climate change for cetaceans are difficult to predict. It has been postulated that long-term changes in physical oceanography, induced by ongoing climate change, will affect primary producers and, through knock-on effects, consumers at every level of the food chain and, especially, apex predators such as many marine mammals^1^. Studies on different temporal scales show that cycles in cetacean strandings are correlated to anomalies in temperature^2^, wind^3^ and continental-scale climate systems^4^, such as the North Atlantic Oscillation (NAO). We present data on the repercussions of abrupt early Holocene climate change for cetaceans living in the Mediterranean Sea and on how humans opportunistically adapted to them, through a study on the site of Grotta dell’Uzzo (Fig. 1) in North West Sicily (Italy).

This cave was occupied sporadically from the Late Pleistocene, but regularly only by Mesolithic hunter-gatherers and Neolithic agro-pastoralists during the whole of the early Holocene (Fig. 2). It is, therefore, a key site for the study of the changes in human culture and subsistence economy that took place at that time in the Mediterranean^5^,^6^,^7^,^8^,^9^,^10^. Studies on the material culture from Grotta dell’Uzzo have demonstrated that its early Holocene occupation was characterized by three main cultural episodes: the Mesolithic (~11,110–8,500 yrs cal. BP), the so-called Mesolithic-Neolithic Transition (~8,770–7,850 yrs cal. BP) and the Neolithic (~8,050–7,130 yrs cal. BP). The lithic technology of the transitional phase has been attributed to the Western Mediterranean blade and trapeze complex of the 9^th^ millennium cal. BP, the last Mesolithic techno-complex in the region^10^,^11^.

Human subsistence during the Mesolithic (Trench F, stratigraphic spits 22–15: ~11,110–8,500 yrs cal. BP) was based mainly on the consumption of the meat of terrestrial herbivores, such as red deer (*Cervus elaphus*), and omnivores, such as wild boar (*Sus scrofa*)^5^,^8^. Bird bones are quite abundant, but only a small proportion of these were introduced to the cave by humans for consumption. Marine resources played a minor role for most of the Mesolithic and their exploitation targeted mainly intertidal rocky shore molluscs^12^, while fishing was extremely limited^5^,^6^. Plant foods probably contributed to the diet more than transpires from the rare vegetal refuse recovered during excavations at Grotta dell’Uzzo, which includes wild legumes, acorns and wild grape^13^. Studies of dental microwear and pathology suggest that the Mesolithic hunter-gatherers had mixed diets, which included regular and relatively high consumption of vegetals^14^. An impoverished and mainly plant-based diet has been hypothesized for the adult female from burial Uzzo 1A, as a result of spectroscopic analysis of a bladder stone^15^.

The Mesolithic-Neolithic ‘transition phase’ was the last period when the subsistence economy was solely based on hunting and gathering (Trench F, stratigraphic spits 14–11: ~8,770–7,850 yrs cal. BP)^5^,^8^. During this brief interlude, fishing increased and the range of species exploited broadened to include aquatic birds, fish, marine turtles and, as demonstrated by the present study, cetaceans.

In the early Neolithic (Neolithic I: Trench F, stratigraphic spits 10–6: ~8,050–7,400 yrs cal. BP) the dietary breadth broadened further and human groups in NW Sicily started exploiting introduced domestic plants and animals, albeit still relying heavily on wild resources^5^,^8^. Hunting of large and medium-sized ungulates retained a prominent role in subsistence and fishing peaked at this time, given that 82% of all fish remains and 75% of all identifiable individuals from Grotta dell’Uzzo were found in early Neolithic contexts^16^. Fishing targeted exclusively species that can be caught from the shore, either year-round or seasonally, and the most exploited taxon was grouper (*Epinephelus* spp.). A few remains of monk seal, *Monachus monachus* (3 elements of which were also present in the last two Mesolithic stratigraphic spits), and a single bone of sea otter, *Lutra lutra*, were recovered from the early Neolithic layer^5^,^8^,^16^. Even in the following Neolithic phase (Neolithic II: Trench F, stratigraphic spit 5–1: ~7,520–7,130 yrs cal. BP) the occupants of the cave exploited, albeit not intensively, a wide range of marine fauna easily-acquired from the coast.

The original aim of this research was to undertake isotope analyses on human and faunal bone collagen from Grotta dell’Uzzo to reconstruct the diet of the hunter-gatherers and early agro-pastoralists who occupied the cave. The results of our isotopic work pushed us to investigate further the zooarchaeology of the site and particularly the cetacean bone assemblage, which will be presented before the geochemical analyses.

## Results

### Cetacean zooarchaeology

The cetacean assemblage from Grotta dell’Uzzo is, to our knowledge, the largest and most diverse of its age (Supplementary Fig. 1), given that sites of similar antiquity have only yielded few bones^17^. A total of 224 Number of Identified Specimens (NISP) attributable to the order Cetacea were recovered almost exclusively in areas of the cave where Mesolithic-Neolithic transition deposits occur (Supplementary Table 1). In Trench F (Supplementary Table 2), the stratigraphically most reliable trench studied in detail, 87% (n = 106) were found in the Mesolithic-Neolithic transition stratigraphic units (F-12, F-13 and F-14). Only 16 remains were recovered from the two units below these and may have moved down the sequence post-depositionally.

The majority of the cetacean bones recovered from Grotta dell’Uzzo are fragmentary and, therefore, difficult to identify to species. Of the NISP attributable to Cetacea (Supplementary Table 1): 24.1% were only identifiable to order, 68.3% are Delphinidae and 7.6% belong to Mysticeti or large Odontoceti (e.g. *Balaeonoptera* sp. or *Physeter macrocephalus*)^5^. The 17 NISP attributable to these large cetacean taxa are not assignable to sub-order morphologically and meaningful estimates of the Minimum Number of Individuals (MNI) cannot be made because they include almost exclusively vertebral and rib fragments (Supplementary Fig. 1). Isotope analyses show that the large cetacean vertebra sampled for our palaeodietary study (S-EVA 25630) likely belongs to a mysticete (possibly a *Balaenoptera*, as discussed in the caption to Supplementary Table 8), given that it is a trophic level lower than the Delphinidae. Estimations of the meat weights represented by the fauna from the Mesolithic-Neolithic transition, presented in the Supplementary Information (section 1; Supplementary Tables 3–5), show that cetaceans were a protein source similar in scale to terrestrial ungulates, but two orders of magnitude greater than fish.

The majority of the cetacean remains from Grotta dell’Uzzo are attributable to Delphinidae (Supplementary Fig. 1; Supplementary Tables 1 and 2), including at least three species^5^: long-finned pilot whale (*Globicephala melas*), Risso’s dolphin (*Grampus griseus*) and short-beaked common dolphin (*Delphinus delphis*). The Delphinidae are represented almost exclusively by undiagnostic vertebral fragments and it is not possible to make meaningful estimates of MNI for the taxa of this family either, which may also include other species resident (i.e. *Stenella coeruleoalba*, *Tursiops truncatus*, *Phocoena phocoena*) and non-resident (e.g. *Pseudorca crassidens*) in the Mediterranean. Only about 5% of cetacean skeletal elements from Trench F and M are bones other than vertebrae, and these include a limb fragment and scapula likely of *Gr. griseus*, two periotic bones of different individuals of Delphinidae, a mandibular fragment of a mysticete or large odontocete and a cranial fragment of an undetermined cetacean.

A taphonomic investigation of the bone surfaces was undertaken to assess whether the Grotta dell’Uzzo hunter-gatherers butchered cetacean carcasses. The specimens were observed using a stereomicroscope (stereo NIKON SMZ 1000) at different magnifications (40x–160x) and a Scanning Electron Microscope (SEM; TM3000, HITACHI) on moulds coated in araldite. This assessment has allowed us to observe lithic tool butchering cut-marks on the articular surfaces of 9 vertebrae or epiphyseal vertebral discs: 3 of Mysticeti or large Odontoceti and 6 of Delphinidae. The position of the cut-marks suggests that they were produced in the process of disarticulating the carcasses. Significant taphonomic signatures have been observed on a caudal vertebra (Fig. 3) and a caudal vertebral disc (Fig. 4) of Delphinidae, as described in the captions to these illustrations. The fact that the most abundant elements are vertebrae (i.e. the anatomical elements with the highest meat yields), and that the taphonomic signatures observed on the cetacean bones are compatible with those present on skeletal remains of cetaceans exploited by humans^18^, testify that carcasses were carved to their core to acquire transportable portions for consumption^19^.

Establishing whether these cetaceans were actively acquired is not straightforward, given that procurement cannot always be distinguished from opportunistic exploitation on zooarchaeological grounds^20^. Had the cetacean bones from Grotta dell’Uzzo belonged to hunted animals, the evidence presented here would constitute the oldest for whaling and/or drive hunting worldwide, given that only stranded animals were exploited earlier^21^,^22^. The earliest, albeit indirect, evidence for whaling is claimed to be the rock art from Bangu-dae in South Korea^23^ and even the oldest possible date for these petroglyphs is later than the Mesolithic-Neolithic transition phase at Grotta dell’Uzzo. The lack of taphonomic signatures of harpooning on the bones and of whaling artifacts at the cave, however, suggest that stranded, rather than hunted, cetaceans were exploited^5^,^8^. Moreover, hunting for the different cetacean taxa recovered at Grotta dell’Uzzo would have required techniques specialized for capturing animals with very different habits, which usually took hundreds, if not thousands, of years to develop^17^,^24^. Whilst in theory some gregarious cetaceans, such as dolphins and pilot whales, can be caught using boats and non-specialized weaponry^25^, fast large marine mammals that lack buoyancy, such as rorqual and sperm whales, cannot^20^. It should also be pointed out, however, that drive hunting of cetaceans such as pilot whales is not a simple task, as it requires profound knowledge of the sea and many boats to encircle the delphinids and drive them towards the shore, which presupposes a high level of organization and large numbers of people^26^. The chase out at sea may last days and it is a highly dangerous activity, which necessitates good navigational skill and adequate boating technology.

There is no concrete evidence for offshore fishing by Mediterranean foragers during the early Holocene^27^. The occurrence of bluefin tuna bones at Mesolithic sites in the eastern Mediterranean and the ability of early Holocene foragers to voyage at sea, as attested by the maritime-borne exchange of obsidian in the Aegean, have been used to claim deep-sea fishing in the Mesolithic. The tuna fish bones from Aegean and Adriatic sites, however, are not numerous and belong to individuals that can be caught using set nets in the vicinity of the coast, as done traditionally till a few decades ago. Solid evidence for regular deep-sea navigation is scant for the central and western Mediterranean before the spread of the Neolithic^28^, which occurred later than the exploitation of cetaceans at Grotta dell’Uzzo.

In the remote possibility that central Mediterranean foragers had mastered the boating technology required to pursue highly mobile and rapid cetaceans, they would also have had to coordinate individuals from different groups all accustomed to using watercraft offshore, in a concerted effort to drive odontocetes towards the shore as in pilot whale drive hunts^26^ for which there is no archaeological support. Moreover, if the last hunter-gatherers of NW Sicily hunted cetaceans, it is unclear why such activity should have been abandoned after its successful development and why it was only much later that evidence for it is available from the Mediterranean^29^,^30^.

### Isotope analyses

**Figure 2:**
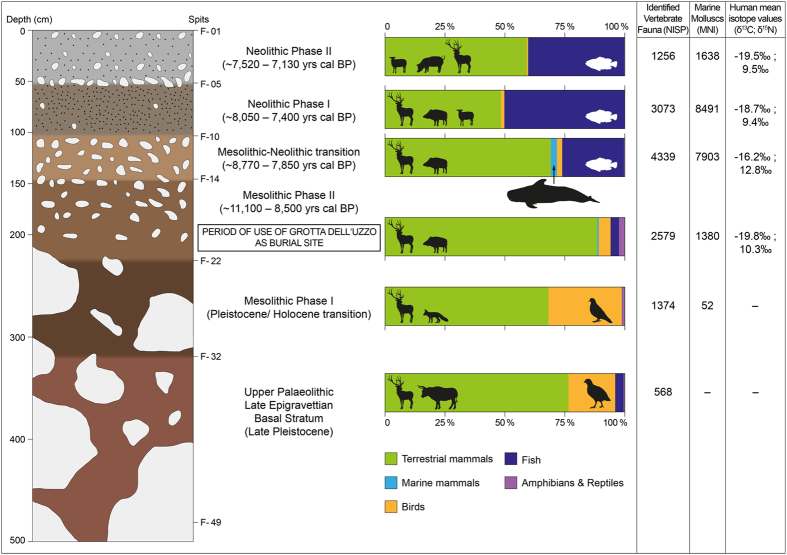
Stratigraphy of Grotta dell’Uzzo with information on the archaeological and isotopic record. Cetacean bones amount to ~2.5% of the vertebrate remains in the Mesolithic-Neolithic transition, constituting the largest assemblage of this antiquity. The pigs represented by the wild boar symbol in ‘Neolithic Phase I’ belong both to the wild and domestic forms. The silhouettes of the main vertebrate faunal taxa were drawn by Marcello A. Mannino.

**Figure 3:**
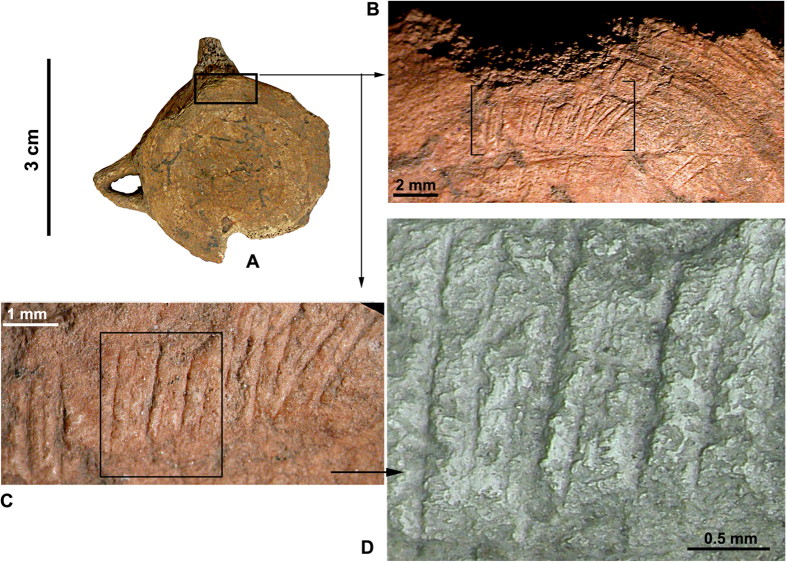
Butchering cut-marks on caudal vertebra of Delphinidae (*Globicephala cf. Gl. melas*). This bone bears cuts on the lateral margin of the cranial side (**A**), near one of the transverse processes, where part of the surface had been removed during separation from the anterior vertebra (**B**). The marks are numerous, short (~2 mm), straight and sub-parallel. Fourteen striations are deep and repeated (**C**; **D**: view under the SEM), with perpendicular edges, while one is oblique, testifying to the inclination of the lithic tool during the cutting action. The semi-circular orientation of these striations follows the curvature of the bone, as it was rotated during dismemberment.

**Figure 4:**
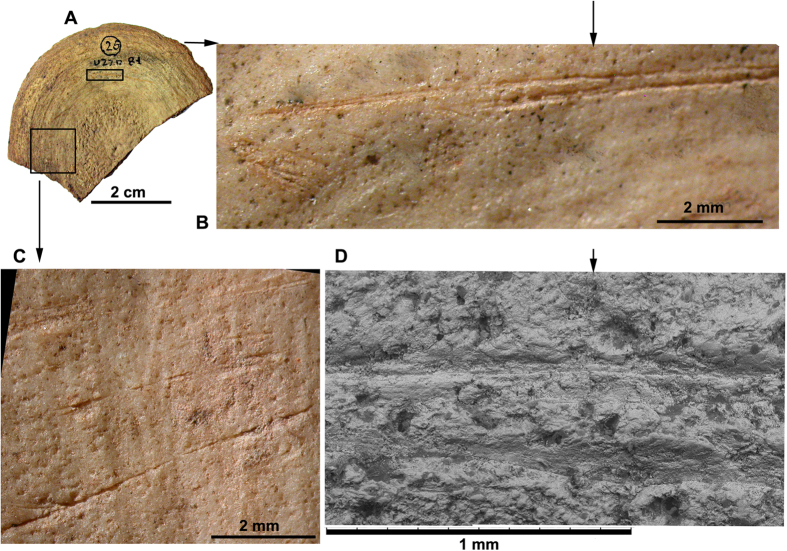
Butchering cut-marks on caudal vertebral disc of Delphinidae (*Grampus cf. Gr. griseus*). This bone (**A**) bears two groups of striations, the first of which (**B**) composed of two long, sub-parallel, and in parts deep grooves, associated with a series of short, superficial and clustered marks, oblique to the former, suggesting perseverance of cutting action in the same part of the vertebra. Under the SEM (**D**), compact and oblique edges are still observable in areas where the lithic tool penetrated deeply, leaving diagnostic secondary striations. The second group of cut-marks (**C**), at least 7 of different length and depth, are at the edge of the vertebral disc and follow its circular shape.

**Figure 5:**
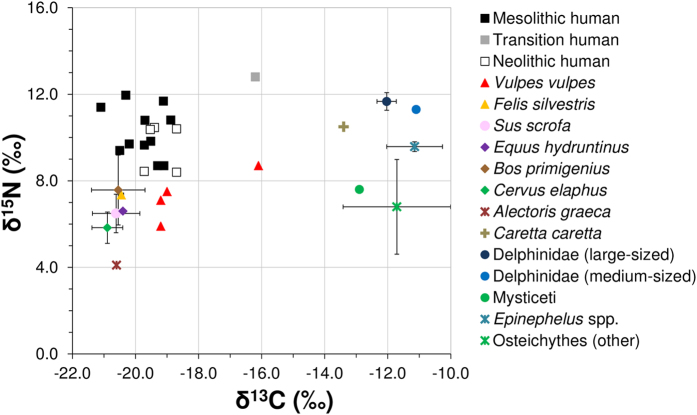
Carbon (δ^13^C) and nitrogen (δ^15^N) isotope composition of collagen from human and animal bones recovered at Grotta dell’Uzzo. Humans have been sampled from all three early Holocene site occupation phases. The terrestrial fauna is from Mesolithic and Mesolithic-Neolithic transition layers, while the marine fauna is mainly from the Mesolithic-Neolithic transition and Neolithic layers (details on the samples are in Supplementary Information section 2 and the data are fully listed in Supplementary Tables 6–8 and 10).

Carbon (δ^13^C), nitrogen (δ^15^N) and sulphur (δ^34^S) isotope analyses on collagen extracted from human and animal bones from Grotta dell’Uzzo were undertaken to investigate the role of marine foods in the diet of its occupants^31^,^32^,^33^,^34^ and to verify whether cetacean meat was consumed (Fig. 5; Supplementary Information: section 2, Fig. 2 and Tables 6–11). Stable isotope analyses of animal tissues are an established method for palaeodietary reconstruction, because their chemical composition reflects that of the food consumed. The isotopic composition of mammalian bone collagen reflects mainly that of the dietary protein consumed, over the turnover time of the analysed tissue. Carbon isotope ratios (δ^13^C) provide data on the photosynthethic pathway (i.e. C_3_, C_4_ or CAM) and/or ecosystem of origin (i.e. terrestrial, freshwater or marine) of the protein consumed^31^. Nitrogen isotope ratios (δ^15^N) reflect the trophic level of the acquired dietary protein and increase by 3–5‰ every step up the food chain^32^,^33^. Sulphur isotope ratios (δ^34^S) provide us data on the biome from which protein was acquired (i.e. terrestrial, freshwater or marine), as well as on mobility^34^. The δ^34^S composition of bone collagen also varies in relation to the geology over which an individual lived during the formation of the analysed tissue and between coastal areas, affected by the so-called sea spray effect, and the hinterland. The δ^34^S isotope ratios in littoral soils and plants living in them are typically higher than those inland, because the sea spray alters the composition by introducing enriched sulphur that makes coastal values intermediate between fully terrestrial and marine ones. Isotopes of this element can, therefore, inform us on whether, during the turnover of the analysed tissue, an individual spent most of its time along the coast or further inland (i.e. in areas unaffected by sea spray).

The δ^13^C values of the terrestrial (= –22.0‰ – –19.0‰) and marine (= –15.1‰ – –9.6‰) fauna conform to the compositions expected for animals living in these two ecosystems (Fig. 5). The terrestrial fauna have carbon isotope compositions typical of animals living in ecosystems characterized by C_3_ vegetation, such as pre-Neolithic Europe^35^,^36^. Moreover, the δ^34^S ratios also clearly distinguish terrestrial from marine animals. As expected, in both biomes, the δ^15^N values reflect the trophic level differences between herbivores, omnivores and carnivores. The only animal with an isotopic composition that does not conform to its customary ecological requirements is a red fox (*Vulpes vulpes*) from the Mesolithic-Neolithic transition (δ^13^C = −16.1‰; δ^15^N = 8.7‰; δ^34^S = 14.8‰), which acquired a high proportion of protein (≥40%) from marine fauna. *V. vulpes* is a generalist predator that uses resources opportunistically according to their availability and feeds on aquatic organisms when they come across their carcasses. Fish are rarely consumed by foxes living in the Mediterranean Basin^37^ and in the rest of Europe^38^. It can, thus, be concluded that such high quantities of marine protein could only have been acquired by the fox that lived during the Mesolithic-Neolithic transition had carcasses of large aquatic animals been plentiful on a yearly basis, as when cetaceans mass strand.

The isotope data indicate that Mesolithic humans (n = 11) consumed little or no marine protein (means: δ^13^C = −19.8 ± 0.7‰; δ^15^N = 10.3 ± 1.1‰; δ^34^S = 8.3 ± 0.0‰). The means for these hunter-gatherers are one trophic level higher (δ^13^C = **~+**1.0‰ and δ^15^N = **~**+4.0‰)^33^ than those of the two main prey species, *C. elaphus* and *S. scrofa*, combined (δ^13^C = −20.8‰ and δ^15^N = 6.2‰). This is in accord with dietary reconstructions based on the faunal food refuse, which show that red deer and wild boar were overwhelmingly the main sources of dietary protein during the Mesolithic^5^,^8^ (Fig. 2). Early Neolithic humans (n = 2) consumed more marine foods than their Mesolithic predecessors (means: δ^13^C = −18.7 ± 0.0‰; δ^15^N = 9.4 ± 0.4‰; δ^34^S = 9.2 ± 1.9‰), in line with the zooarchaeological evidence showing that this was the period of most intense fishing^5^. Overall, however, Neolithic individuals (n = 5; means: δ^13^C = −19.2 ± 0.5‰; δ^15^N = 9.6 ± 1.1‰) relied little on marine resources.

The only human to acquire a substantial proportion of protein from the sea was the individual from the Mesolithic-Neolithic transition (δ^13^C = -16.2‰; δ^15^N = 12.8‰; δ^34^S = 13.4‰). This hunter-gatherer has, in fact, the highest δ^13^C value of any prehistoric Mediterranean human^39^,^40^,^41^, as well as by far the highest δ^15^N and δ^34^S values of any other individual from Grotta dell’Uzzo. Taken together these data indicate that only during the Mesolithic-Neolithic transition seafood contributed notably to human diet at Grotta dell’Uzzo and that this adaptation represents an exception in the context of the Mediterranean during prehistory.

The IsoSource^42^ and FRUITS^43^ mixing models, used for estimating the proportions of protein obtained from the different sources (Supplementary Tables 12 and 13), indicate that the transitional hunter-gatherer acquired 40–49% of protein from marine animals. Cetaceans were the most likely marine sources, accounting for ~32% of such protein, with Delphinidae (i.e. pilot whales and other true dolphins) ranking higher than mysticetes. Overall fish rank lower than cetaceans and grouper contributed less protein than Delphinidae, as highlighted also by estimations of the meat weights represented by the fauna from the Mesolithic-Neolithic transition (Supplementary Tables 3–5). Given that adult human cranial bone collagen turns over in ≥10 years, cetaceans should have been available and consumed yearly to acquire so much meat from them.

### Radiocarbon dating

The AMS radiocarbon dating undertaken for this research was aimed at refining the chronology of the best-preserved stratigraphic sequence at Grotta dell’Uzzo (i.e. Trench F) and of the Mesolithic-Neolithic transition, in particular. The dates produced here (Supplementary Table 14) were used with published ones (Supplementary Table 15) on charcoal^7^ and marine shell^6^ for a Bayesian analysis of Trench F (Supplementary Fig. 3). The analysis, conducted using OxCal 4.2^44^, shows that the different phases of occupation defined by Tagliacozzo^5^,^8^ are chronologically distinct and in succession. The occupation phases had the following durations: Mesolithic II (11,110–9,300 to 8,770–8,500 yrs cal. BP), Mesolithic-Neolithic transition phase (8,770–8,500 to 8,050–7,850 yrs cal. BP), Neolithic I (8,050–7,850 to 7,520–7,400 yrs cal. BP), Neolithic II (7,520–7,400 to 7,470–7,130 yrs cal. BP). This new chronology for Grotta dell’Uzzo demonstrates that the transition lasted no more than 900 years and, possibly, as little as 450 years. This cultural phase was shorter than suggested by previous dating studies^6^,^7^, overlapping significantly with the period of climatic deterioration that, starting from around 8,600 years ago, culminated with the so-called 8,200 years cal. BP (hereafter 8.2-kyr-BP) event^45^.

In addition, to verify whether the isotopic data from the transitional human reflects the proverbial needle in a haystack or, in other words, if this individual’s diet informs us about short-term abundance of cetacean meat, we radiocarbon dated its collagen and that of the two best-preserved Cetacea, a mysticete and a delphinid (Supplementary Information section 3). The dates on the cetaceans and the human that consumed cetacean meat (Supplementary Table 16), suggest that strandings may have concentrated during a narrow interval of around two hundred years (~8,520–8,320 yrs cal. BP), which is the overlap of their modelled calibrated ages. As discussed in detail below in the methods section on calibration and reservoir corrections, the time of the deposition of Sapropel 1 was characterized by higher reservoir ages (ΔR = 149 ± 50 yrs) and a further change in these coincided with the 8.2-kyr-BP event^46^,^47^,^48^. We have not corrected for these higher reservoir age values, given the uncertainties of their entity during the climate changes in question, but account for them by noting that the period when portions of cetaceans were taken back to Grotta dell’Uzzo may have been ~8,520–8,320 yrs cal. BP or less than ~200 yrs after. According to radiocarbon data from climatic records across the North Atlantic region^48^, this overlaps fully with the timing of the 8.2-kyr-BP event (~8,500–8,250 yrs cal. BP). The overlap in the calibrated ages of the human from the Mesolithic-Neolithic transition and the two dated cetaceans (Supplementary Table 16) confirms that large quantities of cetacean flesh were available to local hunter-gatherers at the onset of the 8.2-kyr-BP rapid climate change (RCC).

## Discussion

The cetacean assemblage from Grotta dell’Uzzo is typical of recent death assemblages worldwide, as it is dominated by Delphinidae^49^, which may imply that it was generated by attritional strandings occuring normally through time. However, both the archaeological and isotopic records show that cetaceans were exploited only during the Mesolithic-Neolithic transition, making attritional strandings an unlikely explanation for their presence at the site. In the absence of whaling, the synchronicity between the 8.2-kyr-BP event and the presence of cetaceans at Grotta dell’Uzzo suggest that the last hunter-gatherers of NW Sicily exploited animals that probably stranded due to climate-driven environmental instability. This RCC resulted from the curtailment of the North Atlantic Deep Water (NADW) formation, and its associated northward heat transport, following the catastrophic drainage of the Laurentide lakes into the Atlantic Ocean from 8.47 ± 0.3 kyr cal. BP^45^,^50^. This caused rapid cooling in sea surface temperatures and reductions in sea surface salinities and air temperatures in the circum-North-Atlantic region, as well as changes in precipitation regimes and a drop in atmospheric CO_2_ globally. Temperatures plummeted in Greenland (~7–8 °C) within a couple of decades from the initial meltwater pulse and over 150 ± 30 yrs after the outburst into the Atlantic region north of 30°N and in the Mediterranean Basin^51^. The rate of change was very fast in the first decade after perturbation, slowing down by the third^50^. In the Mediterranean Sea, the 8.2-kyr-BP event coincided with an interruption in the deposition of Sapropel 1^52^, an organic-rich sediment that accumulated during the Holocene Climatic Optimum due to reduced deep water ventilation caused by enhanced freshwater discharge linked to the African monsoon.

Natural mass-stranding death mechanisms include herding behaviour, changes in large-scale oceanic fronts, diseases and harmful algal blooms^1^,^2^,^3^,^4^,^53^,^54^,^55^. Most of these factors, except algal blooms, may have favoured the strandings, because, otherwise, the assemblage would have been dominated by mysticetes^55^ and their flesh would probably not have been consumed. Even at times of relative climatic stability, decadal shifts in oceanic gyres enforce bottom-up controls on marine ecosystems and affect plankton and consumers at every trophic level^53^. These knock-on effects up the food chain, in turn, cause reductions and shifts in prey abundance (i.e. phenotypic mismatching), increasing susceptibility to disease in animals with complicated life-cycles and high feeding requirements, such as cetaceans. Long-term ecological studies have demonstrated that cetacean distribution^53^, strandings and mortality^2^,^3^,^4^ are linked to large-scale climate variability, mediated through its effects on oceanic circulation and prey distribution. These factors were more pronounced during the 8.2-kyr-BP event and the concomitant interruption of Sapropel 1, possibly increasing physiological stress^54^ and resulting in regular mass strandings of odontocetes in the Mediterranean Sea, at least.

This scenario is compatible with the findings of our isotopic palaeodietary study, because, for a human to acquire ~32% of protein from their meat, cetaceans should have been available year-on-year for ≥10 years, given the slow turnover of adult cranial bone collagen. We hypothesize that, after the outburst of the Laurentide lakes (8.47 ± 0.3 kyrs cal. BP), cetacean mass strandings occurred yearly in NW Sicily. Similar frequencies are known at stranding hotspots, where there is strong geographical clustering of mass strandings, involving from two to hundreds of live odontocetes^56^. Hotspots are located in bays with gently-sloping beaches, flanked by steep rocky headlands (Supplementary Information section 4), such as the San Vito lo Capo peninsula, and work as traps because parts of them are acoustical ‘dead zones’, where geometric effects distort echolocation preventing cetaceans in distress from navigating back out at sea^57^.

At the sea level stand of around 8,200 years ago, the Gulf of Castellammare had all the features of stranding hotspots (Supplementary Fig. 4) and its south-western corner near the locality of Scopello, around 5.5 km south of Grotta dell’Uzzo (Supplementary Figs 5 and 6), resembled acoustical dead zones where cetaceans get trapped. Albeit sporadically, Grotta dell’Uzzo was also occupied during the Younger Dryas (Fig. 2), a harsher RCC than the 8.2-kyr-BP event. Around the Pleistocene/Holocene transition, however, sea levels were so low (~75 m below present sea level)^58^ that the bathymetry of a smaller Gulf of Castellammare would have been too steep for a stranding hotspot. This lends support to the hypothesis that mass strandings only took place regularly when the right bathymetric morphology was present at a time of climate-driven environmental change, as in NW Sicily around 8,200 years ago.

Our research has implications for understanding human adaptations to RCC, as well as for studies of cetacean strandings, conservation and genetics. The diet of the latest hunter-gatherer from Grotta dell’Uzzo represents an exception, given that marine resources were not consumed intensively by previous Mesolithic humans at the same site, as well as by prehistoric groups in the Mediterranean, due to the oligotrophy of this sea^39^. Humans and foxes opportunistically exploited the dramatic increase in cetacean strandings linked to climate-driven environmental changes caused by the 8.2-kyr-BP event. We cannot, however, reach definitive conclusions as to whether the hunter-gatherers of NW Sicily were actively opportunistic, driving cetaceans in distress to the shore in a primordial form of drive hunting, or passively exploited annual strandings as other animals would have done.

The impact on cetaceans of rapid climate changes, such as the 8.2-kyr-BP event, may explain some of the low genetic diversity^59^, population declines^60^ and bottlenecks^61^ detected in cetacean mitochondrial DNA, at least in the circum-North-Atlantic region. Cetaceans adapted to regular rates of climate change over shorter (i.e. El Niño) or longer time scales (i.e. the Pleistocene-Holocene deglaciation)^62^, but not to marked yearly changes in climate and oceans that may simply have been too fast for long-lived animals with low fecundity. These probably constitute a threat for cetaceans in the near future, given that the mean cooling around 8,200 years ago was similar in magnitude (~1.8–4.0 °C)^52^,^53^ to the global warming predicted for this century (~1.0–3.7 °C), for which it may represent a worst case scenario^63^. Moreover, like anthropogenic climate change, the 8.2-kyr-BP event also affected salinity, precipitation and atmospheric CO_2_. This is all the more alarming in regions such as the Mediterranean, where cetacean populations are in constant decline and the effects of global warming on them are anticipated to be greatest^64^.

## Methods

### Collagen extraction for isotope analyses

Collagen extraction for carbon, nitrogen and sulphur isotope analyses was undertaken according to the protocols in use at and approved by the laboratories at the Department of Human Evolution, Max Planck Institute for Evolutionary Anthropology in Leipzig (Germany). Bone samples were cleaned by air abrasion and demineralized in 0.5 M HCl at 4 °C. Demineralized bones were then rinsed in deionized water and gelatinized at 70 °C in a pH 3 solution for 48 hours. The insoluble fraction was then filtered, first with 5 mm Ezee^©^ filters and then with >30 kDa Amicon^©^ ultrafilters. The purified solution was then frozen and freeze dried for 48 hours. Stable isotope analyses were performed at the same laboratories on a Thermo Finnigan Flash EA coupled to a Delta Plus XP Isotope Ratio Mass Spectrometer. Stable isotope ratios are expressed using the delta (δ) notation, as part per thousand (‰) difference relative to international standard reference materials. δ^13^C ratios are reported relative to the V-PDB (Vienna Pee Dee Belemnite) standard, δ^15^N ratios to atmospheric N_2_ (AIR) and δ^34^S ratios to the V-CDT (Vienna Canyon Diablo Troilite) standard. The analytical precision based on repeated measurements was better than 0.20‰.

### Materials and quality of collagen for isotope analyses

A total of 70 human bones were sampled for isotope analyses, 57 from buried individuals (all burials were sampled except Uzzo 3) and 13 recovered loose within the deposits (Supplementary Table 6). Only 33 human samples yielded extracts (47.1%), 10 of which are from bones not originating from burials. This is indicative of the poorer state of preservation of the human bones from the burials, only 40.4% of which yielded extracts as opposed to 76.9% of those from the deposits. In addition, all the latter extracts are well-preserved collagen, according to established quality criteria^65^. Two of these have low yields (i.e. S-EVA 8012 and 8014), but we consider them collagen because they meet all other quality criteria. Of the extracts from the buried individuals, 11 had C/N ratios outside of the accepted range for collagen (=2.9–3.6)^66^, so these have been excluded from our study. We consider the remaining 11 extracts well-preserved collagen because they meet all the quality criteria^65^, although 3 have yields of 0.5% (S-EVA 2758, 2760, 2771). Possible reasons for the poorer preservation of inhumated bones are that (i) some may have been contaminated by consolidants used to preserve them, given their high %C (i.e. S-EVA 503, 2773, 7993, 7995), and that (ii) many of the burials were covered only by a thin layer of sediment and, thus, subject to harsher depositional conditions, after historic and prehistoric deposits lying above were progressively removed by shepherds cleaning out the excrements of ovicaprids kept at Grotta dell’Uzzo through the millennia.

A total of 85 animal bones were sampled for isotope analyses in this study, 68 of which from layers attributable to the Mesolithic (including the Mesolithic-Neolithic transition) and 17 from Neolithic deposits (Supplementary Tables 7 and 8). The latter include marine taxa not represented or rare in pre-Neolithic assemblages. Of the animal bones sampled, 71 (=83.5%) yielded extracts that on the basis of elemental analyses (%C, %N, C/N molar ratio) and isotopic composition are compatible with well-preserved collagen^65^. The weight percentages of these yields, however, are variable: 43.7% are >1.0%, 35.2% are >0.5% and 21.1% are <0.5%. For the purposes of our palaeodietary study, we have considered collagen all extracts with yields ≥0.5% and %C, %N and C/N molar ratios that meet the standards proposed by van Klinken^65^. We have, therefore, not taken into consideration the results from the 15 specimens with yields <0.5% for our reconstructions, although they generally have isotopic compositions within the range of variation of well-preserved samples.

For the sulphur isotope analysis (Supplementary Table 9), the data (%S, C/S and N/S molar ratios) from all mammals match the quality criteria established for well-preserved collagen^34^. The fish bone samples have sulphur contents slightly below the proposed range for biogenic collagen and should be treated with care. The isotopic data suggest that there was a loss of sulphur-containing amino-acids in the collagen, which does not seem to have altered its isotope composition.

### Radiocarbon dating

Bone pretreatment methods for AMS radiocarbon dating are those established by Talamo and Richards^67^. The 15 dated extracts meet the quality criteria by van Klinken^65^ for well-preserved collagen (Supplementary Tables 6 and 8), with the exception of two *Homo sapiens* samples, S-EVA 8014 and 8012, which have respectively collagen yields of 0.6% and 0.8%. The extracts from these two individuals meet the other quality criteria for collagen (i.e. %C, %N and C/N) and their radiocarbon dates are compatible with the known chronology of the context of origin. We, thus, consider these samples as well-preserved collagen and have kept them in our dating study. Well-preserved collagen was dated at the Liebniz Laboratory of the Christian Albrechts Universität of Kiel (KIA), at the Klaus Tschira Laboratory of the Curt-Engelhorn-Zentrum Archaeometrie in Mannheim (MAMS) and at the Oxford Radiocarbon Accelerator Unit (OxA).

### Calibration and reservoir corrections

The AMS radiocarbon dates on bone collagen samples are reported in Supplementary Table 14 and calibrated using OxCal 4.2^44^ with the IntCal13 and Marine13 curves^68^, respectively for terrestrial and marine organisms. The calendar ages of the cetaceans, which are known to be migratory, were corrected for the reservoir effect using the correction estimated by Reimer and McCormac^69^ for the Mediterranean Basin (ΔR = 58 ± 85 ^14^C yrs). The reservoir correction adopted for the dates on grouper (*Epinephelus* spp.), a non-migratory fish, was that proposed by Siani *et al.*^70^ for Sicily (ΔR = 71 ± 50 ^14^C yrs). To calibrate the date for the *H. sapiens* from the Mesolithic-Neolithic transition phase (S-EVA 8010) we used both curves. According to the IsoSource 1.3.1 and FRUITS 1.0 mixing models (the application of which is discussed above and in Supplementary Information section 2), this individual consumed approximately 40 to 49% marine protein, so for calibration purposes we estimated in 40 ± 10% the proportion of ^14^C originating from the marine reservoir. We used the reservoir correction estimated for the whole Mediterranean: ΔR = 58 ± 85 ^14^C yrs^69^, because the marine protein was mainly from cetaceans that migrate across this sea. The Mediterranean marine reservoir age was relatively constant for the past 7,000 years, while in the early Holocene and Late Pleistocene ΔR varied considerably and this variation is not straightforward to estimate^47^. In fact, during the deposition of Sapropel 1 between 9,000 and 6,000 years calibrated BP^52^, the reservoir age may have been higher by 149 ± 50 ^14^C yrs than the Holocene average, due to changes in water circulation patterns^46^. Moreover, ^14^C production maxima corresponding to solar output minima, around the 8.2-kyr-BP event, caused sea surface waters to be older by ∼100 yrs^48^, which complicates an accurate estimation of the reservoir effect.

## Additional Information

**How to cite this article**: Mannino, M. A. *et al.* Climate-driven environmental changes around 8,200 years ago favoured increases in cetacean strandings and Mediterranean hunter-gatherers exploited them. *Sci. Rep.* **5**, 16288; doi: 10.1038/srep16288 (2015).

## Supplementary Material

Supplementary Information

## Figures and Tables

**Figure 1:**
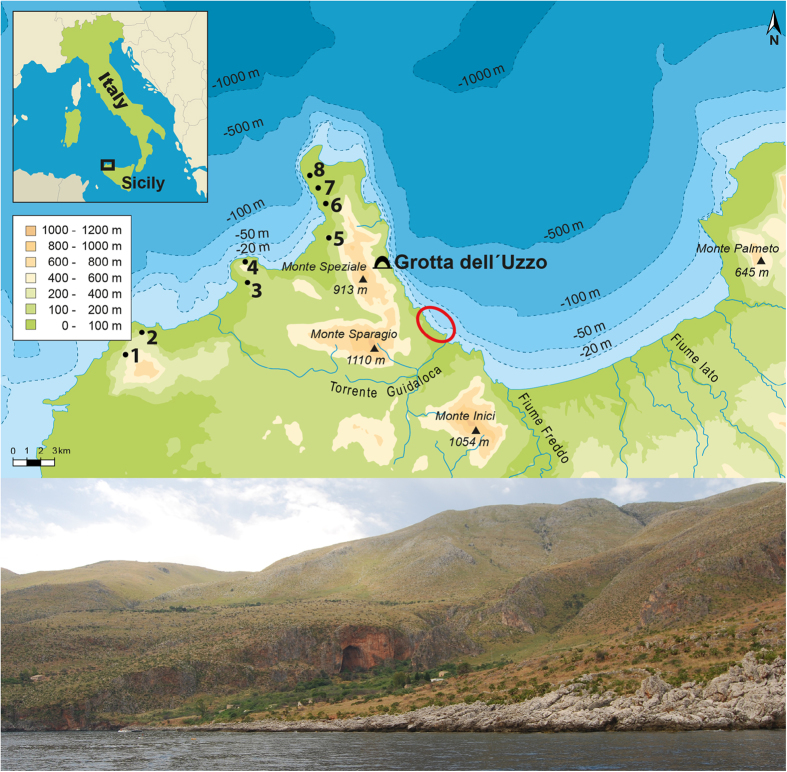
Location of Grotta dell’Uzzo (San Vito lo Capo peninsula) on the Gulf of Castellammare and view of the cave from the north. Caves are present all along the coasts of NW Sicily and some of those containing Upper Palaeolithic and Mesolithic deposits are shown: 1. Grotta Martogna; 2. Grotta Emiliana, Grotta del Maltese; 3. Grotta Mangiapane, Grotta Scurati; 4. Grotta del Crocifisso; 5. Grotta Perciata; 6. Grotta di Mezzo; 7. Grotta dei Cavalli; 8. Grotta di Cala Mancina. The supposed locality of the cetacean strandings (circled in red) coincides with the area around Scopello, as hypothesized by analogy with stranding hotspots worldwide (Supplementary Information section 4). The map was generated using Adobe Illustrator CS. The photo was taken by Marcello A. Mannino.
