# Supplementary material for: Climate-driven environmental changes around 8,200 years ago favoured increases in cetacean strandings and Mediterranean hunter-gatherers exploited them
Source: Sci Rep. 2015 Nov 17;5:16288. doi: 10.1038/srep16288 (PMC4648091; doi:10.1038/srep16288)
Supplement: Supplementary Information [file srep16288-s1.pdf]

Supplementary Information

Climate-driven environmental changes around 8,200 years ago favoured increases in cetacean strandings and Mediterranean hunter-gatherers exploited them

Marcello A. Mannino, Sahra Talamo, Antonio Tagliacozzo, Ivana Fiore, Olaf Nehlich, Marcello Piperno, Sebastiano Tusa, Carmine Collina, Rosaria Di Salvo, Vittoria Schimmenti, Michael P. Richards

Table of contents

1. Cetacean zooarchaeology
   Supplementary Figure 1 2
   Supplementary Table 1 3
   Supplementary Table 2 3
   Supplementary Text: Meat weight estimates 4
   Supplementary Table 3 6
   Supplementary Table 4 7
   Supplementary Table 5 7

2. Isotope analyses
   Supplementary Text: Faunal isotope composition 8
   Supplementary Text: Human isotope composition 9
   Supplementary Figure 2 12
   Supplementary Table 6 13
   Supplementary Table 7 14
   Supplementary Table 8 15
   Supplementary Table 9 16
   Supplementary Table 10 16
   Supplementary Table 11....................................17
   Supplementary Text: Mixing models for human diets 17
   Supplementary Table 12 21
   Supplementary Table 13 22

3. Radiocarbon dating
   Supplementary Table 14 23
   Supplementary Table 15 24
   Supplementary Table 16 24
   Supplementary Figure 3 25

4. Location and number of cetacean strandings
   Supplementary Text 26
   Supplementary Figure 4 29
   Supplementary Figure 5 30
   Supplementary Figure 6 31

Supplementary Information References 32

1. Cetacean zooarchaeology

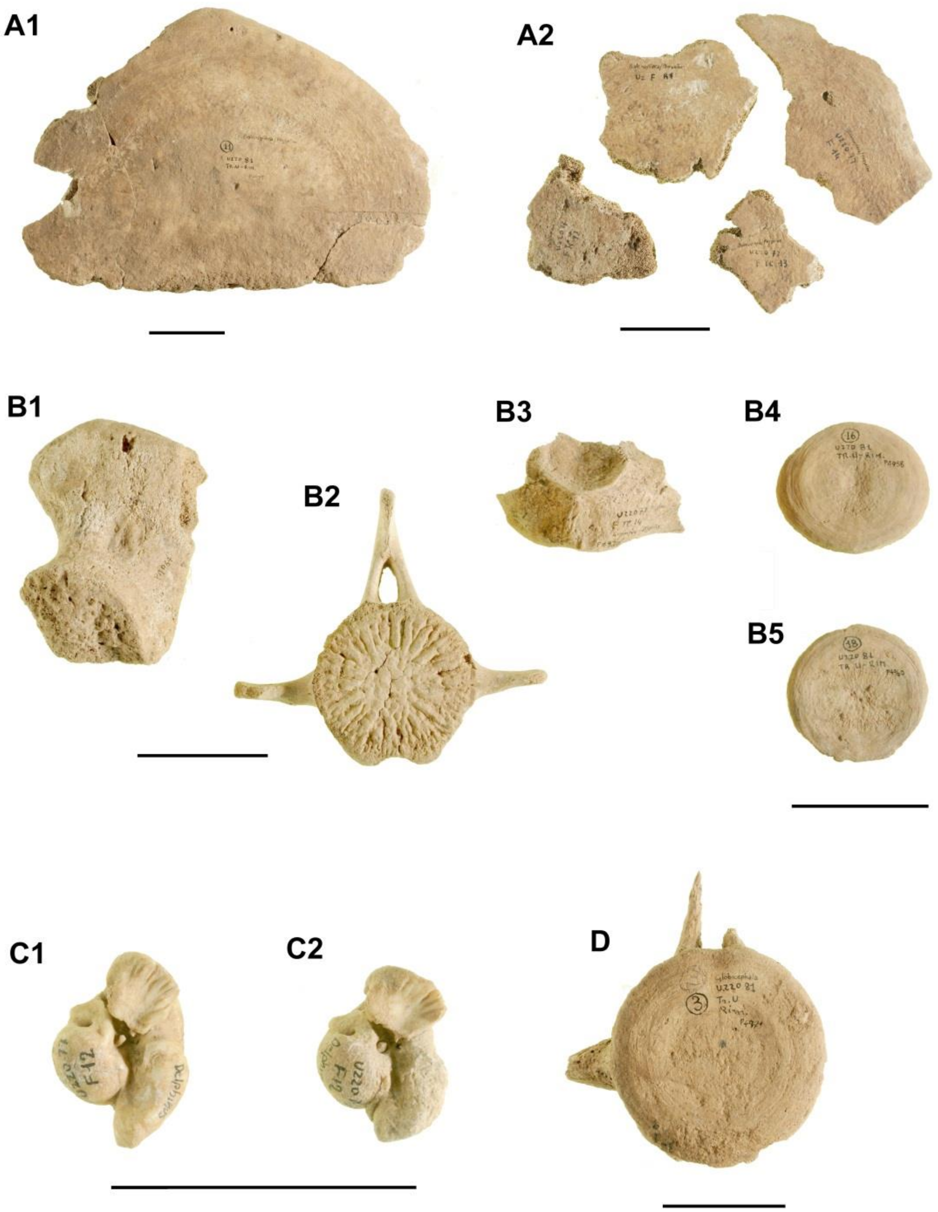

Supplementary Figure 1 │ Selection of cetacean bones recovered at Grotta dell'Uzzo. A. Mysticeti or large Odontoceti: A1. vertebral disc (Trench U), A2. vertebral disc fragments (Trench F spits 11-14); B. Delphinidae (*Grampus* cf. *Gr. griseus*): B1. ulna (Trench F spit 14), B2. lumbar vertebra (Trench F spit 14), B3. atlas (Trench F spit 14), B4-B5. vertebral discs (Trench U); C. Delphinidae: C1-C2. right periotic bones (Trench F spit 12); D. Delphinidae: vertebra (Trench U). The scale bars are 5 cm.

Supplementary Table 1 │ Number of Identified Specimens attributed to the order Cetacea recovered from different trenches excavated at Grotta dell'Uzzo

taxon	trench							total
	D	F	M	P	U	X	Y	
Mysticeti or large Odontoceti	-	14	1	-	2	-	-	17
Delphinidae	41	61	8	2	36	3	2	153
Cetacea (undetermined)	1	47	5	-	1	-	-	54
Cetacea (total)	42	122	14	2	39	3	2	224

Supplementary Table 2 │ Number of Identified Specimens attributed to the order Cetacea from Trench F

taxon	Mesolithic-Neolithic transition			Mesolithic II		total
	F-12	F-13	F-14	F-15	F-16	
Mysticeti or large Odontoceti	9	1	4	-	-	14
Delphinidae	8	15	30	1	7	61
Cetacea (undetermined)	13	15	11	1	7	47
Cetacea (total)	30	31	45	2	14	122

According to the interpretation of the stratigraphy following the excavation, spits F-12 to F-14 are within the Mesolithic-Neolithic Transition phase, while F-15 and F-16 are in the uppermost levels of the Mesolithic II phase.
Based on the original identifications by Pier Francesco Cassoli published by Antonio Tagliacozzo[1,2], the bones we classify as 'Mysticeti or large Odontoceti' may have belonged to *Balaenoptera* sp. (rorqual whale) and/or *Physeter macrocephalus* (sperm whale). Isotope analyses suggest that at least one of these specimens (a vertebral fragment; S-EVA 25630 in Supplementary Table 8) is attributable to a baleen whale (Mysticeti), given that its diet was typical of a filter-feeder compatible with a *Balaenoptera* sp. The only member of this genus with a resident population in the Mediterranean Sea is *Balaenoptera physalus* (fin whale)[3]. On morphological grounds, however, it cannot be excluded outright that some of the specimens classified here as 'Mysticeti and large Odontoceti' actually do not belong to large cetaceans that at present have no viable populations and have very rarely been observed in the Mediterranean Sea, such as *Megaptera* (humpback whale) and *Eubalaena* (North Atlantic right whale)[3]. According to the original identifications, the most abundant Delphinidae are *Globicephala melas* (pilot whale), followed by *Grampus griseus* (Risso's dolphin) and *Delphinus delphis* (short-beaked common dolphin), all of which at present have resident populations in the Mediterranean[3].

Meat weight estimates

The fragmentary state of the cetacean assemblage from Grotta dell'Uzzo prevents us from attempting a detailed estimation, through Meat Utility Indices (MUI) or Food Utility Indices (FUI), of the quantity of meat represented by the bones taken back to the cave. Utility indices are formulated to quantify the amount of meat obtainable from different anatomical portions of vertebrates[4,5]. These indices are used to estimate the proportions of meat available and to interpret the archaeological record of human butchering, transport and consumption decisions. These estimates can be misleading, however, when comparing animals of very different size, as when determining the dietary potential of fish versus cetaceans. Cetacean remains, as those of large terrestrial mammals such as elephants, are under-represented or even 'invisible' archaeologically, because large portions of flesh can be taken back to home bases without leaving traces of their presence, especially when elements with high MUI were targeted[6].

MUI for odontocetes have been developed by Savelle and Friesen[5] on a harbour porpoise (*Phocoena phocoena*). The cetacean assemblage from Grotta dell'Uzzo contains almost exclusively remains of vertebrae[1,2], which are the anatomical elements with the highest MUI[5]. Crania and other anatomical elements with low food utility indices (e.g. scapula, sternum, flipper) might have been left behind at the stranding location, where probably most of the butchery took place. This represents further evidence that the bones in question are the remains of cetacean portions taken back to the cave for consumption. Traditional hunter-gatherers often took bones with low MUI back to sites for use in rituals, in the production of artifacts or in building as architectural elements[7], all of which can probably be discarded in the case of Grotta dell'Uzzo. The few bones from the site with low MUI were probably introduced incidentally, as so-called riders or due to inexperience in butchering animals not previously exploited by the hunter-gatherers of NW Sicily.

For the aims of this study it is sufficient to highlight the difference in the scale of animal protein available when large cetaceans and delphinids stranded, compared to that represented by the fish and terrestrial mammals exploited by the occupants of Grotta dell'Uzzo. For this purpose, we have calculated ballpark estimates of the meat weights for grouper (*Epinephelus* spp.) and other fish from the different occupation phases at the site (Supplementary Table 3)[1,2,8]. The limitations of the cetacean bone assemblage, discussed above, force us to simply report the known weights of the different large cetacean and delphinid taxa likely represented at Grotta dell'Uzzo (Supplementary Table 4) and crudely account for the weight of their skeletons using the MUI developed for odontocetes[5]. Again for broad comparative purposes we report the body weights of red deer (*C. elaphus*)[9] and wild boar (*S. scrofa*)[10], as well as the weights corrected for bone weight (Supplementary Table 5). In doing so, we are aware that the weight of consumable tissues in mammals is considerably lower than the weight of the body without the bone weight, given for example that only about 50% of a deer is actually edible[11]. However, for our purposes it is sufficient to highlight the difference in scale of edible tissues obtainable from the different taxa. The comparison of the meat represented by terrestrial and marine taxa from the Mesolithic-Neolithic transition shows that: 1. the total meat weight estimated for fish from the transition (= 180.2kg) is less than that of a mean-sized *Gr. griseus* (~375kg); 2. Delphinidae could have provided similar proportions of meat to those obtainable from tens of individuals of *C. elaphus* and *S. scrofa* (i.e. in the order of thousands of kilograms of consumable tissues) had their strandings been regular and included large taxa resident in the Mediterranean, such as *Gl. melas*.

Adding Mysticeti or large Odontoceti to the calculations, the amount of cetacean protein available would have been vastly superior to that from fishing or even hunting. The late hunter-gatherer from Grotta dell'Uzzo obtained cetacean protein mainly from Delphinidae (Supplementary Tables 1 and 2), which also likely reflects the rarer strandings of rorquals. Nevertheless, the difference in scale between

the protein represented by all the fish remains compared to that available by exploiting simply the cetaceans recovered from the cave emerges even taking the lowest possible number of Mysticeti or large Odontoceti (1 MNI) and Delphinidae (3 MNI = 1 *D. delphis*, 1 *Gl. melas*, 1. *Gr. griseus*) individuals. The carcasses of these animals would have offered roughly 30,000 kg of edible meat, two orders of magnitude higher than what fishing offered throughout the occupation of the site. Given that cetacean protein would have been available only for a brief interlude of, at most, a few decades, then the reason for the difference between a fish bone assemblage accumulated over hundreds of years and a short term abundance of large cetacean and delphinid meat becomes apparent.

Supplementary Table 3 │ Gross meat weight estimates for fish from Grotta dell'Uzzo

Trench F phase (stratigraphical spits)	grouper MNI	grouper NISP	weight largest grouper (kg)	weight mean grouper (kg)	(i) grouper weight from MNI [MNI*mean] (kg)	(ii) grouper weight from NISP [NISP%* 7.48kg] (kg)	(iii) total weight [MNIweight + NISPweight/2 + (weight of other fish)] (kg)
Neolithic II (F-05 – F-01)	47 (36.4%)	1071 (45.7%)	6.3	1.9	89.3	341.8	237.0 (21.4)
Neolithic I (F-10 – F-06)	36 (27.9%)	739 (31.5%)	3.3	1.1	39.6	235.6	195.0 (57.4)
Mesolithic-Neolithic (F-14 – F-11)	36 (27.9%)	486 (20.7%)	14.8	4.3	154.8	154.8	180.2 (25.4)
Mesolithic (F-22 – F-15)	10 (7.8%)	50 (2.1%)	5.8	3.0	30.0	15.7	27.0 (4.1)
TOTAL	129	2346	14.8	2.6	313.7	747.9	639.2

The calculations of meat weights represented by the fish from Grotta dell'Uzzo have been made using published zooarchaeological data[1,2,8]. We estimated the meat weight that might have been available during each phase: (i) for grouper based on MNI counts; (ii) for grouper based on NISP percentages; (iii) for all ichthyofauna by adding to the mean of estimates (i) and (ii) the estimate of the weight for fish other than grouper for each phase. To calculate meat weight based on NISP we used estimate (i) for the phase with the highest mean grouper size (i.e. the Mesolithic-Neolithic Transition) and assumed that 154.8kg of grouper meat are equal to 20.7% of 'grouper NISP'. We, therefore, divided 154.8kg by 20.7% to calculate how much meat 1% of 'grouper NISP' would equate to (= 7.48kg). This value was then multiplied by the percentage of 'grouper NISP' for each phase to obtain estimate (ii). This estimation is based on the assumption that NISP counts reflect the proportion of fish taken to the site, rather than degree of fragmentation. This may have resulted in an overestimation of the fish weights of the Neolithic phases and an underestimation of that for the Mesolithic, which if one takes solely into account the higher estimate (i) may represent a marginally higher total weight (= 34.1kg). For our calculations of total fish weight not to be affected by the deficiencies of the two quantification methods (i.e. MNI and NISP) we produced an average of estimates (i) and (ii) and then added the weight for other fish present in each phase. The estimate for the weight of the other fish (shown below total weight in (iii) was calculated dividing the grouper weight based on NISP by the NISP% of grouper from the same layer and then multiplying the result by the NISP% of the other fish. Our calculations are likely overestimates, given that they are based on grouper, which is a larger fish than most others exploited by the occupants of Grotta dell'Uzzo. Overall, it is clear that the fish weight for each phase would have been in the order of hundreds rather than thousands of kilograms.

Supplementary Table 4 │ Body weights of the cetacean taxa from Grotta dell'Uzzo

Species	female	male	mean (corrected for bone weight)
Common dolphin	70-100 kg	80-110 kg	90 kg
Delphinus delphis			(84 kg)
Risso's dolphin	350 kg	400 kg	375 kg
Grampus griseus			(352 kg)
Pilot whale	1,800-2,500 kg	3,000-3,500 kg	2,700 kg
Globicephala melas			(2,535 kg)
Sperm whale	15,000-24,000 kg	45,000-57,000 kg	35,300 kg
Physeter macrocephalus			(30,782 kg)
Fin whale	30,000-120,000 kg		75,000 kg
Balaenoptera physalus			(65,400 kg)

Data on weights was obtained from the website of the American Cetacean Society (http://acsonline.org) and from: http://www.marinemammalscience.org/index.php?option=com_content&view=article&id=418&Itemid=279. The values in brackets provide a ballpark estimate for body weight without bones, calculated by subtracting 6.1% from the total weight for the odontocetes and 12.8% for the mysticete according to what is discussed by Savelle and Friesen[5] on Meat Utility Indices for cetaceans.

Supplementary Table 5 │ Body weights for the main terrestrial ungulates exploited during the Mesolithic-Neolithic transition

species	weight (mean)	MNI phase	upper value estimate (corrected for bone weight)	mean value estimate (corrected for bone weight)
Red deer	70-250 kg	38	9,500 kg	7,030 kg
C. elaphus	(185 kg)		(8,692 kg)	(6,432 kg)
Wild boar	50-200 kg	25	5,000 kg	3,125 kg
S. scrofa	(125 kg)		(4,675 kg)	(2,922 kg)

Body weight data are from *Ungulati delle Alpi*[9] for *C. elaphus* and *The Encyclopedia of Mammals*[10] for *S. scrofa*. Corrections for bone weights are for wild boar, the one obtained by Rowly-Conwy et al. (= 6.5%)[4], and for red deer, the minimum bone weight for mammals in general (= 8.5%) proposed by Casteel[12], given that we did not find a specific calculation for this ungulate species. The mean value estimates are likely more accurate, although still overestimates given the relatively small size of the skeletal remains of both these taxa at Grotta dell'Uzzo[1,2].

2. Isotope analyses

Faunal isotope composition. Terrestrial herbivores have mean $\delta^{13}C$ ratios around -20.0‰, ranging between -20.9‰ (*C. elaphus*) and -20.5‰ (*B. primigenius*) (Supplementary Table 7), typical of animals living in biogeographic regions, such as southern Europe, where C_3 plants are dominant. As expected, terrestrial carnivores have slightly higher mean $\delta^{13}C$ ratios: -20.5‰ in the case of *F. silvestris* and -19.1‰ in the case of all *V. vulpes* excluding the outlying individual (S-EVA 8737 = -16.1%) that scavenged on stranded cetaceans. The $\delta^{15}N$ ratios of terrestrial mammals reflect the differences in trophic levels between species (Supplementary Table 7), with the carnivores (*V. vulpes* and *F. silvestris*) having higher values than the omnivores (*S. scrofa*) and herbivores (*C. elaphus*). Two taxa do not fit this pattern (i.e. *E. hydruntinus* and *B. primigenius*), but these are only present in the earliest Mesolithic levels and their values probably reflect different environmental conditions, which produced higher $\delta^{15}N$ baseline in the late Pleistocene and early Holocene. The $\delta^{34}S$ ratios also vary as expected (Supplementary Table 9), given that they clearly distinguish terrestrial (7.9-13.4‰) from marine fauna (15.3-17.2‰). The sulphur isotope composition of marine faunal bone collagen from Grotta dell'Uzzo, however, is noticeably lower than that of seawater (~20‰), but within the ranges for modern and archaeological marine mammals and fish (for a review see Nehlich et al.[13]). Of the terrestrial fauna, only foxes have intermediate $\delta^{34}S$ ratios, one possibly because it had lived along the coast in habitats affected by sea spray (S-EVA 8736), while the other also because it consumed whale meat (S-EVA 8737). The latter is the only animal with an unexpected $\delta^{13}C$ ratio for its habitual habitat, having consumed a similar proportion of marine protein to the human from the same phase (~40-49%).

The isotopic composition of the marine fauna from Grotta dell'Uzzo is compatible with that expected of animals living in marine ecosystems. In fact, pelagic fish (~-13.3‰), mammals (-12.9‰ to -11.1‰) and reptiles (-13.4‰) have lower $\delta^{13}C$ ratios than rocky shore coastal taxa (~-11.1‰), while fish

spending part of their life in brackish water habitats have values intermediate between terrestrial and fully marine (-15.1‰ to -10.0‰). Our analyses on the largest prehistoric sample of well-identified Mediterranean marine animals studied to date, therefore, do not confirm observations by Vika and Theodoropolou[14], who suggested that fish in the Mediterranean may have 'terrestrial-like' $\delta^{13}C$ ratios. For instance, an individual attributed to Serranidae, a fully marine family which includes grouper, was claimed by Vika and Theodoropolou[14] to have $\delta^{13}C$ values as low as -19.2‰, a discrepant result that might be explained through misidentification. The carbon isotope composition of the bone collagen of marine fauna from Grotta dell'Uzzo is similar to that of analogous animals living in oceanic environments, albeit marginally higher[15]. The largest difference relative to animals living in oceans, such as cetaceans along the Atlantic coast of NW Africa, is that nitrogen isotope ratios are lower by ~1.0-3.0‰ in the Mediterranean, due to the lower $\delta^{15}N$ of phytoplankton and particulate organic nitrogen (PON) produced by N_2 fixation and N recycling in this enclosed sea[16].

Human isotope composition. The Mesolithic humans have mean $\delta^{13}C$ values of -19.8‰ and $\delta^{15}N$ values of -10.2‰ (Supplementary Table 6), respectively ~0.8‰ and ~3.5‰ higher than the herbivores (i.e. *E. hydruntinus*, *B. primigenius* and *C. elaphus*) and ~0.8‰ and ~3.7‰ higher than the omnivores (i.e. *S. scrofa*). The means of the isotopic values for the Mesolithic humans are one trophic level higher ($\delta^{13}C$ = +1.0‰ and $\delta^{15}N$ = +4.0‰) than the means of the two main prey species, *C. elaphus* and *S. scrofa*, combined ($\delta^{13}C$ = -20.8‰ and $\delta^{15}N$ = 6.2‰). This is in accord with dietary reconstructions based on the faunal food refuse, which show that red deer and wild boar were overwhelmingly the main source of dietary protein consumed by Mesolithic hunter-gatherers[1,2]. This also matches the results of a previous isotopic study undertaken on only two of the Mesolithic humans buried at Grotta dell'Uzzo[17]. Neolithic humans have similar isotopic compositions; the Mann-Whitney U test shows their carbon (p. 0.145) and nitrogen (p. 0320) isotope values not to be significantly different from those of the

Mesolithic humans. Nevertheless, the mean $\delta^{13}C$ value of the Neolithic individuals taken together is slightly higher (= -19.2‰) and mean $\delta^{15}N$ value lower (= 9.6‰) than those of their Mesolithic predecessors. This suggests that the meat of terrestrial mammals was their main source of protein, but that they consumed less of it and/or their diet may have included a small proportion of low trophic level marine resources. It is noteworthy (although on the basis of little data) that the two earliest Neolithic individuals (S-EVA 8013 and 8772) have a higher mean $\delta^{13}C$ and lower mean $\delta^{15}N$ values (-18.7±0.0‰; 9.4±0.4‰) than Mesolithic (-19.8±0.7‰; 10.3±1.2‰) and later Neolithic (-19.5±0.2‰; 9.8±1.2‰) ones. The early Neolithic individuals may, thus, have consumed higher proportions of seafood than Mesolithic and late Neolithic ones, compatibly with the faunal data which shows that they lived at the time of most intensive fishing at Grotta dell'Uzzo[1,2]. Overall, the small role of marine protein in Mediterranean diets may be largely attributable to the oligotrophy of Mediterranean Sea and to the late development of technologies adequate for its intensive exploitation[18].

The hunter-gatherer from the Mesolithic-Neolithic transition has the highest $\delta^{13}C$ value (= -16.2‰) of any prehistoric human from the Mediterranean. The $\delta^{15}N$ value (= 12.8‰) and $\delta^{34}S$ value (= 13.4‰) are the highest for humans at Grotta dell'Uzzo. These isotopic values indicate that the transitional human acquired a high proportion of dietary protein from high trophic level marine fauna, as well as from terrestrial sources such as *C. elaphus* and *S. scrofa*. According to the mixing models, described below, this individual consumed approximately between 40 to 49% of marine protein, mostly from Delphinidae. This late hunter-gatherer has a $\delta^{34}S$ ratio intermediate between terrestrial and marine fauna (Supplementary Table 9), similar to one of the foxes from the same period and indicative of a difference in diet linked either to a coastal bias in territoriality or to a greater consumption of marine foods. Studies of the seasonality of resource exploitation at Grotta dell'Uzzo[1,2,19] suggest that, from the end of the Mesolithic to the Neolithic, the cave was occupied during every season and that there was no change in territoriality. The high $\delta^{34}S$ value of the transitional hunter-gatherer, thus, likely reflects

consumption of marine foodstuffs only available during the transition, such as cetacean meat, rather than a greater susceptibility to the so-called sea spray effect.

In an isotopic study of early Neolithic humans from south-east Italy, Lelli et al.[20] suggested that 5-30% of protein consumed by coastal groups originated from marine foods and that one individual from Grotta delle Mura ($\delta^{13}C$ = -17.7‰) acquired 25-50% of such protein. Lelli et al.[20] correctly pointed out that, if collagen carbon was also derived from carbohydrates and lipids (both of which are depleted relative to protein), the degree of marine consumption would be underestimated. However, the upper estimate exaggerates the contribution by marine foods to the diets of the Neolithic humans in question, because the marine endpoint used in their mixing models is too low ($\delta^{13}C$ = -14.5‰ instead of -9.0‰), as demonstrated by data presented in this and previous studies[14,18]. This endpoint was obtained averaging the values of four unidentified fish defined as ‘marine’, which may include taxa from different marine habitats (e.g. coastal and pelagic waters, brackish habitats such as lagoons and/or estuaries) not all of which have high $\delta^{13}C$ values. In conclusion, the human from the Mesolithic-Neolithic transition at Grotta dell’Uzzo is to date the Mediterranean hunter-gatherer who consumed the highest proportion of marine protein. This exception reflects the high proportion of cetacean meat that became available following the unusual stranding frequencies that occurred in the run up to the 8.2-kyr-BP event.

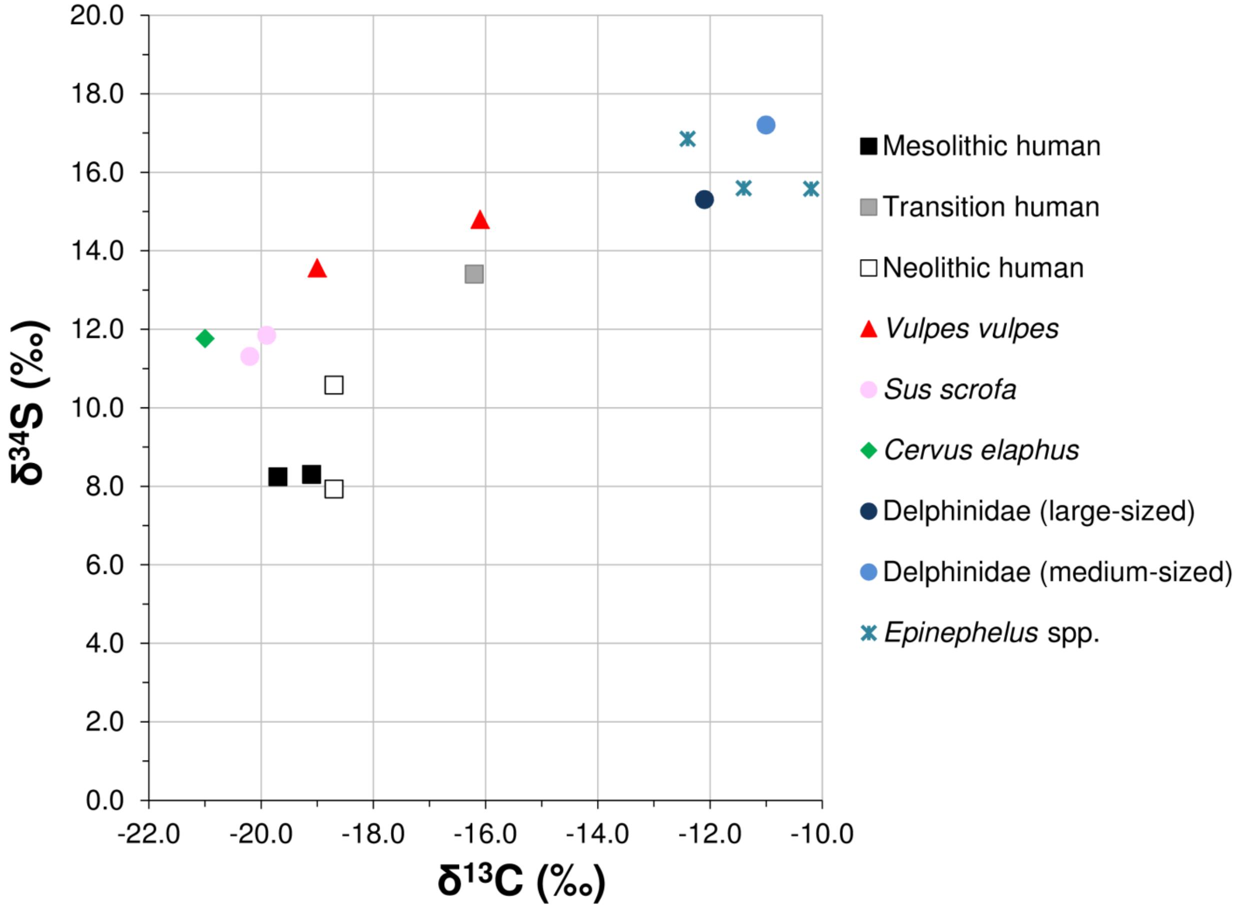

Supplementary Figure 2 │ Carbon ($\delta^{13}C$) and sulphur ($\delta^{34}S$) isotope composition of collagen from human and animal bones recovered in Mesolithic and Mesolithic-Neolithic transition layers at Grotta dell'Uzzo. Humans have been sampled from the different phases of site occupation (Supplementary Tables 6 and 9). The terrestrial fauna is from Mesolithic and Mesolithic-Neolithic transition layers (Supplementary Tables 7 and 9), while the marine fauna is from the Mesolithic-Neolithic transition layer (Supplementary Tables 8 and 9).

Supplementary Table 6 │ Carbon ($\delta^{13}C$) and nitrogen ($\delta^{15}N$) isotope values of bone collagen extracted from 34 prehistoric human remains recovered at Grotta dell'Uzzo

laboratory number (S-EVA)	context (gender of adult or age of immature individuals)	element	$\delta^{13}C$ (‰)	$\delta^{15}N$ (‰)	%C	%N	C:N	% yield
2750	*Burial Uzzo 1A (female)*	*rib*	*-20.0*	*10.0*	*15.3*	*4.8*	*3.7*	*0.7*
7990	Burial Uzzo 1A (female)	rib	-20.1	9.4	34.9	12.0	3.4	1.7
7991	Burial Uzzo 1A (female)	rib	-20.3	9.9	43.1	14.1	3.6	2.1
7993	*Burial Uzzo 1B (male)*	*rib*	*-21.0*	*9.2*	*49.4*	*13.9*	*4.2*	*3.0*
15135	Burial Uzzo 1B (male)	rib	-20.5	9.4	11.1	3.7	3.5	1.0
2773	*Burial Uzzo 2 (male)*	*femur*	*-21.0*	*11.1*	*47.2*	*13.2*	*4.2*	*0.9*
502	*Burial Uzzo 4A (male)*	*rib*	*-21.4*	*10.7*	*43.6*	*12.4*	*4.1*	*1.7*
503	*Burial Uzzo 4B (female)*	*rib*	*-22.3*	*10.7*	*58.1*	*13.6*	*5.0*	*1.1*
7995	*Burial Uzzo 4B (female)*	*rib*	*-22.8*	*9.8*	*54.1*	*10.0*	*6.3*	*5.0*
7996	*Burial Uzzo 4B (female)*	*rib*	*-21.2*	*8.5*	*27.8*	*6.7*	*4.8*	*2.3*
504	*Burial Uzzo 5 (male)*	*rib*	*-20.4*	*11.6*	*40.3*	*12.6*	*3.7*	*1.3*
2756	*Burial Uzzo 5 (male)*	*rib*	*-21.8*	*11.7*	*21.8*	*6.7*	*3.8*	*0.8*
7998	Burial Uzzo 5 (male)	rib	-20.9	11.4	45.2	15.0	3.5	5.8
7999	Burial Uzzo 5 (male)	rib	-21.2	11.3	40.2	13.5	3.5	3.6
2755	Burial Uzzo 6 (4-5 year old)	rib	-19.5	9.8	42.7	15.1	3.3	2.3
2757	Burial Uzzo 6 (4-5 year old)	vertebra	-19.5	9.7	42.3	15.1	3.3	2.2
505	*Burial Uzzo 7 (male)*	*rib*	*-20.6*	*11.8*	*36.0*	*11.5*	*3.7*	*1.9*
2758	Burial Uzzo 7 (male)	phalanx	-20.3	12.0	42.6	14.2	3.5	0.5
8000	Burial Uzzo 8 (female)	rib	-19.3	8.7	34.3	12.9	3.1	1.1
2760	Burial Uzzo 9 (2-3 month old)	rib	-19.1	11.7	41.3	13.8	3.5	0.5
2761	Burial Uzzo 9 (2-3 month old)	rib	-19.0	11.6	28.7	10.0	3.3	2.1
8009	Burial Uzzo 9 (2-3 month old)	rib	-19.1	11.5	41.0	15.0	3.2	6.5
2771	Burial Uzzo 11 (male)	rib	-19.1	11.7	42.7	14.8	3.4	0.5
8006	*Burial Uzzo 11 (male)*	*rib*	*-19.4*	*9.1*	*17.2*	*5.1*	*3.9*	*2.0*
8014	Burial Uzzo X (male)	ulna	-19.7	10.8	41.4	14.7	3.3	0.6
8012	Trench F Spit16 (adult)	cranium	-19.7	9.7	38.8	13.8	3.3	0.8
8010	Trench F Spit12 (adult)	cranium	-16.2	12.8	40.7	15.4	3.1	1.4
8013	Trench F Spit 9 (adult)	cranium	-18.7	8.4	43.4	15.9	3.2	3.2
2777	Trench H Spit 1 (adult)	humerus	-18.9	10.8	43.4	15.2	3.3	1.8
8773	Trench Y Spit 3 (adult)	cranium	-19.1	8.7	45.8	16.3	3.3	1.0
8772	Trench X Spit 21 (adult)	metatarsal	-18.7	10.4	38.6	14.0	3.2	1.3
2779	Trench L Spit 1 (juvenile)	humerus	-19.5	10.4	43.8	15.1	3.4	1.3
2774	Trench S Spit 1 (adult)	femur	-19.7	8.4	43.1	15.2	3.3	1.3
2778	Trench W Spit 2 (juvenile)	humerus	-19.4	10.5	41.1	14.1	3.4	1.3

Human bones sampled are from individuals recovered in burials (all Mesolithic) and from the deposits of Trench H, F, L, S, W, X and Y. Burials Uzzo 1 to 9 were unearthed during the excavation campaigns which took place from 1975 to 1983[21]. Burial Uzzo 11 was discovered during the excavation campaign of 2004[22], while Uzzo X are the remains of a burial uncovered after digging by a wild boar (*S. scrofa*) in the search for underground storage organs. Burial Uzzo 3 (4-6 month-old infant) was not sampled for curatorial concerns, given that it has been preserved intact for exhibition at the Museo Archeologico Regionale 'Antonino Salinas' in Palermo. Three ribs were sampled from burial Uzzo 10 (S-EVA 8003-8005), but did not yield extracts. The fragments found loose in the deposit include Mesolithic (S-EVA 8012, 2777, 8773), Mesolithic-Neolithic Transition (S-EVA 8010) and Neolithic (S-EVA 8013, 8772, 2779, 2774, 2778) individuals. All human bone fragments recovered loose in the deposits were AMS radiocarbon dated (Supplementary Table 14), with the exception of S-EVA 2779 that comes from Neolithic deposits in Trench L. Specimens with values in italics yielded extracts not compatible with well-preserved collagen.

Supplementary Table 7 │ Carbon ($\delta^{13}C$) and nitrogen ($\delta^{15}N$) isotope values of bone collagen extracted from 43 specimens of prehistoric terrestrial fauna recovered at Grotta dell'Uzzo

laboratory number (S-EVA)	trench & spit	species	element	$\delta^{13}C$ (‰)	$\delta^{15}N$ (‰)	%C	%N	C:N	% yield
8738	F-16	*Vulpes vulpes*	radius	-19.2	5.9	35.2	12.6	3.3	0.7
8736	F-13	*Vulpes vulpes*	radius	-19.0	7.5	42.1	14.8	3.3	1.3
8737	F-13	*Vulpes vulpes*	radius	-16.1	8.7	40.0	14.2	3.3	1.1
8739	F-13	*Vulpes vulpes*	radius	-19.2	7.1	43.2	16.0	3.2	2.5
8741	F-12	*Felis silvestris*	scapula	-20.5	7.3	43.0	15.9	3.2	2.0
8742	F-12	*Felis silvestris*	femur	-20.4	7.4	41.7	14.8	3.3	0.6
2788	A-19	*Sus scrofa*	mandible	-20.0	8.2	42.9	15.3	3.3	4.5
2796	A-12	*Sus scrofa*	astragalus	-20.3	5.9	42.5	15.3	3.2	3.1
2797	A-12	*Sus scrofa*	astragalus	-20.3	6.0	40.6	14.6	3.2	4.3
2795	A-09	*Sus scrofa*	astragalus	-20.3	6.8	39.3	14.3	3.2	4.5
14086	F-24	*Sus scrofa*	ulna	-19.7	6.9	29.4	10.2	3.4	0.8
14084	F-18	*Sus scrofa*	phalanx	-21.8	7.1	42.8	14.0	3.6	0.8
14085	F-18	*Sus scrofa*	phalanx	-20.9	5.7	42.1	13.7	3.6	0.3
8749	F-16	*Sus scrofa*	ulna	-20.4	5.8	39.0	13.1	3.5	0.3
8750	F-16	*Sus scrofa*	ulna	-20.2	6.1	38.0	13.4	3.3	0.9
8754	F-12	*Sus scrofa*	ulna	-19.9	6.6	41.0	14.1	3.4	0.7
8755	F-12	*Sus scrofa*	ulna	-19.9	7.0	42.2	14.4	3.4	0.3
14080	F-12	*Sus scrofa*	phalanx	-21.5	5.7	31.2	10.3	3.5	0.6
14082	F-12	*Sus scrofa*	phalanx	-21.7	7.1	28.9	9.9	3.4	0.8
14083	F-12	*Sus scrofa*	phalanx	-21.0	4.9	34.1	11.4	3.5	0.8
2782	A-21	*Cervus elaphus*	metatarsus	-20.1	5.8	43.0	15.2	3.3	2.3
2781	A-19	*Cervus elaphus*	metatarsus	-21.3	4.8	43.2	15.3	3.3	2.3
2783	A-16	*Cervus elaphus*	metatarsus	-20.4	6.4	42.8	15.6	3.2	2.7
2784	A-16	*Cervus elaphus*	metatarsus	-21.5	6.3	41.2	15.0	3.2	2.2
2785	A-16	*Cervus elaphus*	metatarsus	-21.0	5.3	42.8	15.6	3.2	2.9
14087	F-24	*Cervus elaphus*	phalanx	-21.3	6.6	24.4	8.2	3.5	0.8
8762	F-18	*Cervus elaphus*	scapula	-22.0	6.3	45.6	15.0	3.5	0.1
8763	F-18	*Cervus elaphus*	scapula	-21.0	6.5	43.2	14.9	3.4	0.7
8764	F-18	*Cervus elaphus*	humerus	-21.1	5.2	41.9	14.1	3.5	0.1
19860	F-12	*Cervus elaphus*	metatarsus	-20.6	5.0	36.3	12.5	3.4	1.1
2787	A-21	*Bos primigenius*	metatarsus	-19.6	9.5	43.5	15.1	3.4	2.7
2786	A-03	*Bos primigenius*	metatarsus	-21.6	8.2	42.7	15.6	3.2	3.3
14061	F-18	*Bos primigenius*	metatarsus	-20.4	6.8	45.3	14.7	3.6	0.5
8745	F-17	*Bos primigenius*	scapula	-22.0	7.0	42.4	14.0	3.5	0.1
8743	F-16	*Bos primigenius*	rib	-20.9	7.0	37.3	12.6	3.5	0.1
8744	F-16	*Bos primigenius*	scapula	-20.8	7.5	29.5	9.8	3.5	0.2
14049	F-14	*Bos primigenius*	cranium	-20.6	5.8	16.7	5.6	3.5	0.6
2798	A-21	*Equus hydruntinus*	phalanx	-20.4	6.6	41.4	14.9	3.2	1.9
19871	F-08	*Alectoris graeca*	tibiotarsus	-20.6	4.1	42.6	14.9	3.3	8.1

All specimens, except one (S-EVA 19871), are terrestrial mammals from Mesolithic (A-21 to A-03 and F-24 to F-15) and Mesolithic-Neolithic transition (F-14 to F-11) deposits. S-EVA 19871 is a rock partridge (*Alectoris graeca*) from the Neolithic I phase (F-10 to F-06).

Supplementary Table 8 │ Carbon ($\delta^{13}C$) and nitrogen ($\delta^{15}N$) isotope values of bone collagen extracted from 32 specimens of prehistoric marine fauna recovered at Grotta dell'Uzzo

laboratory number (S-EVA)	trench & spit	taxon	element	$\delta^{13}C$ (‰)	$\delta^{15}N$ (‰)	%C	%N	C:N	% yield
2213	F-27	*Epinephelus* sp.	vertebra	-10.4	9.7	38.1	13.7	3.2	0.8
2278	F-15	*Epinephelus* sp.	vertebra	-11.4	9.2	41.3	14.6	3.3	0.6
2281	F-14	*Epinephelus* sp.	vertebra	-12.4	9.8	40.4	14.7	3.2	1.0
2280	F-12	*Epinephelus* sp.	vertebra	-11.4	9.6	42.5	15.3	3.2	1.6
2214	F-11	*Epinephelus* sp.	vertebra	-10.2	9.5	41.4	14.6	3.3	1.1
2282	F-10	*Epinephelus* sp.	vertebra	-10.7	11.5	43.3	14.7	3.4	0.4
2215	F-09	*Epinephelus* sp.	vertebra	-10.5	10.9	36.4	13.4	3.2	1.5
2283	F-06	*Epinephelus* sp.	vertebra	-10.8	10.3	41.0	14.4	3.3	0.6
2284	F-05	*Epinephelus* sp.	vertebra	-11.1	9.9	41.2	14.4	3.3	0.8
2216	F-04	*Epinephelus* sp.	vertebra	-9.6	10.2	36.2	13.4	3.1	1.3
2285	F-02	*Epinephelus* sp	vertebra	-9.8	9.5	37.3	13.7	3.2	1.9
19865	F-10	*Muraena helena*	vertebra	-11.0	10.0	41.4	14.8	3.3	0.9
19866	F-09	*Muraena helena*	premaxillar	-10.4	8.2	47.1	16.4	3.4	0.7
19867	F-07	*Muraena helena*	vertebra	-13.1	8.2	41.9	13.6	3.6	0.6
19868	F-10	*Diplodus sargus*	dentary	-10.3	6.3	41.2	14.6	3.3	2.4
19869	F-08	*Sparus aurata*	vertebra	-11.1	3.9	39.2	14.2	3.2	0.8
19870	P-06	*Sarpa salpa*	vertebra	-10.8	3.6	41.8	15.2	3.2	1.8
17139	U-mix	*Dentex* sp.	vertebra	-13.4	7.0	36.2	12.7	3.3	1.2
19863	F-18	Mugilidae	vertebra	-15.1	6.5	45.1	15.5	3.4	1.4
19864	F-08	Mugilidae	vertebra	-10.0	4.3	38.0	13.8	3.2	0.6
2790	U-mix	Carangidae	vertebra	-10.3	9.7	33.8	12.0	3.3	1.5
17136	U-mix	Carangidae	vertebra	-11.5	10.1	39.5	13.8	3.3	0.1
17135	U-mix	Scombridae	parasphenoid	-13.3	7.1	32.7	11.4	3.4	0.6
8771	X-19	*Caretta caretta*	plastron	-13.4	10.5	40.1	14.0	3.3	0.5
25630	F-12	Mysticeti	vertebra	-12.9	7.6	32.0	11.7	3.2	5.4
25631	U-mix	Delphinidae (medium)	vertebra	-11.1	11.3	44.1	16.5	3.1	13.6
8770	F-16	Delphinidae (large)	vertebra	-11.8	12.0	43.8	15.4	3.3	0.4
8769	F-15	Delphinidae (large)	vertebra	-12.3	11.6	39.9	13.6	3.4	0.6
8768	F-14	Delphinidae (large)	vertebra	-12.7	11.4	33.8	11.3	3.5	0.3
15934	F-14	Delphinidae (large)	vertebra	-12.8	11.0	32.6	11.3	3.4	0.3
8767	F-13	Delphinidae (large)	vertebra	-11.7	12.1	41.3	14.3	3.4	0.6
15933	F-13	Delphinidae (large)	vertebra	-12.1	11.3	38.3	13.4	3.3	0.6

Marine fauna from Mesolithic (F-27 to F-15; U-mix), Mesolithic-Neolithic transition (F-14 to F-11) and Neolithic (F-10 to F-01; P-06; X-19) deposits. The animals sampled include: grouper (*Epinephelus* sp., mainly attributable to *Epinephelus marginatus*)[8], Mediterranean moray (*M. helena*), white seabream (*D. sargus*), gilt-head bream (*S. aurata*), Sarema porgy (*S. salpa*), common dentex (*Dentex* sp.) and taxa belonging to the family Mugilidae (mullets), Carangidae (jacks, runners and scads), Scombridae (mackerels, tunas and bonitos), loggerhead sea turtle (*C. caretta*), a large cetacean (probably a mysticete on isotopic grounds, as discussed below), a medium-sized Delphinidae (compatible with *Grampus griseus* or *Delphinus delphis*) and vertebral fragments of large-sized Delphinidae (compatible with *Globicephala* cf. *Gl. melas*). The isotope analyses have allowed us to establish that the large cetacean vertebra sampled for our palaeodietary study (S-EVA 25630) probably belongs to a Mysticeti, given that it is a full trophic level lower than the Delphinidae. The original attribution to *Physeter macrocephalus* can be rejected because, similarly to other odontocetes in the Mediterranean, sperm whales feed mainly on cephalopod species *Todarodes sagittatus* and *Histioteuthis bonnellii*[23]. S-EVA 25630 may be a fin whale (*Balaenoptera physalus*), as this is the only resident mysticete in the Mediterranean Sea[3]. In the Ligurian Sea it feeds mainly on northern krill (*Meganyctiphanes norvegica*), which is low down in the food chain[24]. Moreover, S-EVA 25630 has a $\delta^{15}N$ ratio (= 7.6‰) similar to the mean of those of modern *Balaenoptera physalus* specimens from the western Mediterranean basin (= 7.6 ± 0.7‰)[24].

Supplementary Table 9 │ Sulphur ($\delta^{34}S$) isotope values of bone collagen extracted from 15 human and faunal specimens from Mesolithic and Mesolithic-Neolithic transition layers at Grotta dell'Uzzo

laboratory number (S-EVA)	trench & spit	Species	$\delta^{34}S$ (‰)	%S	C/N	C/S	N/S
8012	F-16	*Homo sapiens*	8.2	0.14	3.3	633	225
8773	Y-03	*Homo sapiens*	8.3	0.19	3.3	551	196
8010	F-12	*Homo sapiens*	13.4	0.18	3.1	517	196
8013	F-09	*Homo sapiens*	10.6	0.20	3.2	496	182
8772	X-21	*Homo sapiens*	7.9	0.19	3.2	464	168
2281	F-14	*Epinephelus* sp.	16.8	0.31	3.2	298	108
2280	F-12	*Epinephelus* sp.	15.6	0.36	3.2	270	97
2214	F-11	*Epinephelus* sp.	15.6	0.36	3.3	263	93
15933	F-13	Delphinidae (large-sized)	15.3	0.15	3.3	584	204
2563	U-mix	Delphinidae (medium-sized)	17.2	0.20	3.1	504	189
8736	F-13	*Vulpes vulpes*	13.6	0.20	3.3	481	169
8737	F-13	*Vulpes vulpes*	14.8	0.18	3.3	508	180
8750	F-16	*Sus scrofa*	11.3	0.16	3.4	543	191
8754	F-12	*Sus scrofa*	11.8	0.16	3.3	586	201
8763	F-18	*Cervus elaphus*	11.8	0.15	3.4	658	227

Human specimens are from Mesolithic (S-EVA 8012 and 8773), Mesolithic-Neolithic transition (S-EVA 8010) and Neolithic (S-EVA 8013 and 8772) layers. Terrestrial fauna are from Mesolithic (S-EVA 8750 and 8763) and Mesolithic-Neolithic transition (S-EVA 8736, 8737 and 8754) layers. Marine fauna are all from the Mesolithic-Neolithic transition layer.

Supplementary Table 10 │ Mean carbon ($\delta^{13}C$) and nitrogen ($\delta^{15}N$) isotope values of bone collagen for different taxa from Grotta dell'Uzzo

Taxon	n.	$\delta^{13}C$ (‰)	± 1σ	$\delta^{15}N$ (‰)	± 1σ
Mesolithic humans	11	-19.8	0.7	10.3	1.2
Transition human	1	-16.2	-	12.8	-
Neolithic humans	5	-19.2	0.5	9.6	1.1
Epinephelus spp. (grouper)	5	-11.1	0.9	9.6	0.2
Osteichythes (other)	11	-11.7	1.7	6.8	2.2
Caretta caretta (loggerhead sea turtle)	1	-13.4	-	10.5	-
Alectoris graeca (rock partridge)	1	-20.6	-	4.1	-
Mysticeti	1	-12.9	-	7.6	-
Delphinidae (medium-sized)	1	-11.1	-	11.3	-
Delphinidae (large-sized)	4	-12.0	0.3	11.7	0.4
Vulpes vulpes (red fox)	4	-18.4	1.5	7.3	1.2
Felis silvestris (wild cat)	2	-20.5	0.1	7.4	0.1
Sus scrofa (wild boar)	11	-20.6	0.8	6.5	0.9
Cervus elaphus (red deer)	8	-20.9	0.5	5.8	0.7
Bos primigenius (aurochs)	4	-20.5	0.8	7.6	1.6
Equus hydruntinus (European ass)	1	-20.4	-	6.6	-

The $\delta^{13}C$ and $\delta^{15}N$ values were used for Fig. 4, with the exception of the humans and the red fox presented as single values to show intra-specific variability (Supplementary Tables 6 and 7).

Supplementary Table 11 │ Mean carbon ($\delta^{13}C$), nitrogen ($\delta^{15}N$) and sulphur ($\delta^{34}S$) isotope values of bone collagen for taxa and individuals on which sulphur isotope analyses have been undertaken

Taxon	n.	$\delta^{13}C$ (‰)	± 1σ	$\delta^{15}N$ (‰)	± 1σ	$\delta^{34}S$ (‰)	± 1σ
Mesolithic humans	2	-19.4	0.4	9.2	0.7	8.3	0.1
Transition human	1	-16.2	-	12.8	-	13.4	-
Neolithic humans	2	-18.7	0	9.4	1.4	9.2	1.9
Epinephelus spp. (grouper)	3	-11.3	1.1	9.6	0.2	16.0	0.7
Delphinidae (medium-sized)	1	-11.1	-	11.3	-	17.2	-
Delphinidae (large-sized)	1	-12.1	-	11.3	-	15.3	-
Vulpes vulpes (red fox)	2	-17.6	1.5	8.1	0.6	14.2	0.6
Sus scrofa (wild boar)	2	-20.1	0.2	6.4	0.4	11.6	0.4
Cervus elaphus (red deer)	1	-21.0	-	6.5	-	11.8	-

Mixing models for human diets. To establish the likely proportion of marine protein in the diet of the Mesolithic-Neolithic transition hunter-gatherer (S-EVA 8010), we applied two mixing models: IsoSource 1.3.1[25] and FRUITS 1.0[26]. The application of mixing models was essential to estimate the relative contribution of marine foods in the diet for radiocarbon calibration and reservoir correction. It also proved useful to rank the major sources of protein, both for the transitional human and for the Mesolithic hunter-gatherers from Grotta dell'Uzzo. These models were applied to the Neolithic humans only to establish the proportion of terrestrial versus marine protein, given that the detailed reconstruction of the diet of these individuals is not the object of this paper. The mixing models could not be applied to the *V. vulpes* from the Mesolithic-Neolithic transitional layer with a mixed diet (S-EVA 8737), because suitable end-members are not available.

The application and interpretation of mixing models is not devoid of problems, especially in archaeological case studies. Archaeological applications are problematic because it is difficult to include all potential sources of dietary protein ('end-members') that may have contributed to the mass balance diet represented by the isotopic composition[27]. Mixing models should be applied sparingly in the reconstruction of past diets and only for those questions that they are robust enough to address, for example the proportion of marine versus terrestrial foods when highest and lowest end-members have

been adequately defined. These models may also provide additional insights when comparing major food groups, rather than single sources of protein that might skew the reconstruction. In other words, mixing models may be helpful in estimating the relative contribution of major food sources, if these have been accurately identified archaeologically and used in the calculations[26].

In applying the mixing models we aimed to verify which of the main terrestrial and marine sources likely contributed most dietary protein. Taking into account the findings of the zooarchaeological study[1,2] we have, therefore, assumed that the main sources of animal dietary protein would have been: wild boar (*S. scrofa*), aurochs (*B. primigenius*), red deer (*C. elaphus*), large-sized Delphinidae (e.g. *Globicephala* cf. *Gl. melas*), medium-sized Delphinidae (e.g. *Grampus griseus* or *Delphinus delphis*), Mysticeti (e.g. *Balaenoptera* sp.), grouper (*Epinephelus* spp.), and other fish (Supplementary Tables 10 and 11). Red fox (*V. vulpes*) was not included because taphonomic studies suggest that, as other carnivores, it may not have been consumed regularly[1,2].

The IsoSource mixing model was designed to estimate the distribution of source contributions based on isotopic constraints when the number of sources precludes a unique solution[25] and to determine the range of possible diets that may have resulted in the composition measured in the human tissues. To run the IsoSource model, $\delta^{13}C$ values were corrected by +1.0‰[28] and $\delta^{15}N$ values by +4.0‰[29], the mean increases produced by a trophic level shift. Increments were set at 1% and the tolerance at 0.05, the lowest settings to yield solutions. The program calculated 260,265 mass balance diets for the Mesolithic humans and 28,603 for the transitional individual, the results of which are summarized in Supplementary Table 12. The diets of the Mesolithic hunter-gatherers of Grotta dell'Uzzo may have included almost exclusively terrestrial protein (~96%). In line with the palaeodietary reconstructions based on the faunal remains[1,2], the diet of these humans was dominated by red deer (~54%), with a large input from wild boar (~27%) and a significant one from aurochs (~15%). The hunter-gatherer

from the Mesolithic-Neolithic transition layer had a more mixed diet with terrestrial and marine protein contributing more equal proportions (~60% versus ~40%). According to this model, amongst terrestrial preys, aurochs was the most important source of protein (~35%), at odds with the faunal evidence that suggests that *Bos primigenius* may have been extirpated by the end of the Mesolithic. Of the marine resources, the meat of all cetaceans made by far the most significant protein contribution (~32%), while fish contributed only ~8%. Large Delphinidae account for ~17% of protein and constitute the second largest single source. If one runs the model considering large and medium-sized Delphinidae as a single source, taking into account uncertainties in identification, the contribution of these taxa may have been even higher (~37%). Similarly running the model without aurochs, taking into account its low numbers in the faunal assemblage, wild boar would have been the most important terrestrial source (~41%) followed by red deer (~16%), while Delphinidae altogether would amount again to ~37%. We also ran the model with all three isotopes, but do not consider the results from these analyses accurate, because they do not include all the potential main sources of protein, contrary to those on $\delta^{13}C$ and $\delta^{15}N$ values. The models including $\delta^{34}S$ values suggest that the Mesolithic humans may have obtained ~26% of protein from marine foods, whilst the transitional hunter-gatherer ~58% of protein from the sea and as much as ~39% from Delphinidae alone. To sum up all the models run with the IsoSource 1.3.1 software suggest that cetaceans accounted for at least ~32% of all protein consumed by the Mesolithic-Neolithic transition hunter-gatherer.

The Bayesian mixing model FRUITS (Food Reconstruction Using Isotopic Transferred Signals) was developed to provide estimates of the relative intake of potential food sources by taking into account inherent uncertainties not accounted for by other models[26]. In applying this model we were, therefore, able to include uncertainties for trophic level shifts ($\delta^{13}C = 1.5 \pm 0.5‰$; $\delta^{15}N = 4.0 \pm 1.0‰$; $\delta^{34}S = -0.5 \pm 1.5‰$) and standard deviations for the mean isotope values of each taxon. The outputs of the FRUITS

Bayesian mixing model are presented in Supplementary Table 13. Overall, the results are in accord with those generated by the IsoSource model, although the contributions of each source are slightly different. In the case of the Mesolithic hunter-gatherers, based on $\delta^{13}C$ and $\delta^{15}N$ values, the contribution of marine foods is higher (~18%) than that indicated by the IsoSource model (~4%). However, the model based on $\delta^{13}C$, $\delta^{15}N$ and $\delta^{34}S$ values also suggests a low contribution of marine protein to the diet of these humans (~2.5%).

The FRUITS model based on the $\delta^{13}C$ and $\delta^{15}N$ values suggests that terrestrial and marine foods contributed equal proportions of dietary protein (respectively ~51% versus ~49%) to the nutrition of the Mesolithic-Neolithic transition human. According to this model, *S. scrofa* was the main terrestrial source of protein (~29%), followed by *C. elaphus* (~16%) and *B. primigenius* (~5%), while ~32% and ~17% of marine protein may have been obtained respectively from cetaceans and from fish. The single most important marine source were large-sized Delphinidae (~14%), which together with medium-sized Delphinidae account for ~24% of protein, while the meat of mysticetes may have contributed ~8%. Lower trophic level fish may have provided more protein (~15%) than *Epinephelus* spp. (~2%). Running the FRUITS model considering large and medium-sized Delphinidae as a single source did not yield results.

The FRUITS model run with all three isotopes (i.e. $\delta^{13}C$, $\delta^{15}N$ and $\delta^{34}S$) suggests a similar ranking of the marine resources with cetaceans (i.e. all Delphinidae) contributing ~27% and *Epinephelus* spp. ~12%. However, because the sulphur isotope analyses have been undertaken on fewer individuals of fewer potential source taxa, which would tend to produce more averaged-out estimations, we take the results of the FRUITS model run with $\delta^{13}C$ and $\delta^{15}N$ values as the most accurate estimates, which suggest that ~32% of protein may have originated from cetaceans.

In conclusion, both the IsoSource 1.3.1 and FRUITS 1.0 models indicate that the meat of cetaceans probably contributed about one third (~32%) of the dietary protein of the human from the Mesolithic-Neolithic transition at Grotta dell'Uzzo.

Supplementary Table 12 │ IsoSource 1.3.1 mass balance data for humans from the Mesolithic and Mesolithic-Neolithic Transition based on mean $\delta^{13}C$ and $\delta^{15}N$ values (Supplementary Table 10) and $\delta^{13}C$, $\delta^{15}N$ and $\delta^{34}S$ values (Supplementary Table 11)

Taxon	proportional contribution to diet		
	1st percentile	99th percentile	mean (%)
**Mesolithic humans ($\delta^{13}C$, $\delta^{15}N$)**			
Sus scrofa (wild boar)	0	86.0	26.8
Bos primigenius (aurochs)	0	45.0	15.4
Cervus elaphus (red deer)	3.0	92.0	54.0
Delphinidae (large-sized)	0	4.0	0.8
Delphinidae (medium-sized)	0	4.0	0.7
Mysticeti	0	5.0	0.9
Epinephelus spp. (grouper)	0	4.0	0.7
Osteichythes (other)	0	4.0	0.8
**Mesolithic-Neolithic Transition human ($\delta^{13}C$, $\delta^{15}N$)**			
Sus scrofa (wild boar)	0	49.0	14.4
Bos primigenius (aurochs)	2.0	61.0	35.5
Cervus elaphus (red deer)	0	36.0	9.8
Delphinidae (large-sized)	0	37.0	17.1
Delphinidae (medium-sized)	0	32.0	11.1
Mysticeti	0	16.0	3.9
Epinephelus spp. (grouper)	0	22.0	5.5
Osteichythes (other)	0	11.0	2.7
**Mesolithic humans ($\delta^{13}C$, $\delta^{15}N$, $\delta^{35}S$)**			
Sus scrofa (wild boar)	0	82.0	37.8
Cervus elaphus (red deer)	0	82.0	35.6
Delphinidae (large-sized)	0	35.0	10.5
Delphinidae (medium-sized)	0	23.0	7.1
Epinephelus spp. (grouper)	0	30.0	8.9
**Mesolithic-Neolithic Transition human ($\delta^{13}C$, $\delta^{15}N$, $\delta^{35}S$)**			
Sus scrofa (wild boar)	0	70.0	20.7
Cervus elaphus (red deer)	0	70.0	20.7
Delphinidae (large-sized)	0	69.0	19.9
Delphinidae (medium-sized)	0	66.0	19.2
Epinephelus spp. (grouper)	0	67.0	19.4

Supplementary Table 13 │ FRUITS 1.0 mass balance data for humans from the Mesolithic and Mesolithic-Neolithic Transition based on mean $\delta^{13}C$ and $\delta^{15}N$ values (Supplementary Table 10) and $\delta^{13}C$, $\delta^{15}N$ and $\delta^{34}S$ values (Supplementary Table 11)

Taxon	proportional contribution to diet		
	**2.5th percentile**	**97.5th percentile**	**mean (%)**
**Mesolithic humans ($\delta^{13}C$, $\delta^{15}N$)**			
Sus scrofa (wild boar)	1.0	68.0	26.0
Bos primigenius (aurochs)	1.0	63.0	22.0
Cervus elaphus (red deer)	2.0	80.0	34.0
Delphinidae (large-sized)	0	12.0	3.0
Delphinidae (medium-sized)	0	11.0	3.0
Mysticeti	0	13.0	4.0
Epinephelus spp. (grouper)	0	11.0	3.0
Osteichythes (other)	0	13.0	4.0
**Mesolithic-Neolithic Transition human ($\delta^{13}C$, $\delta^{15}N$)**			
Sus scrofa (wild boar)	29.0	29.0	29.0
Bos primigenius (aurochs)	5.0	5.0	5.0
Cervus elaphus (red deer)	16.0	16.0	16.0
Delphinidae (large-sized)	10.0	10.0	10.0
Delphinidae (medium-sized)	14.0	14.0	14.0
Mysticeti	8.0	8.0	8.0
Epinephelus spp. (grouper)	2.0	2.0	2.0
Osteichythes (other)	15.0	15.0	15.0
**Mesolithic humans ($\delta^{13}C$, $\delta^{15}N$, $\delta^{35}S$)**			
Sus scrofa (wild boar)	43.2	98.7	92.5
Cervus elaphus (red deer)	0	5.5	5.0
Delphinidae (large-sized)	0	3.2	0.9
Delphinidae (medium-sized)	0	2.6	0.7
Epinephelus spp. (grouper)	0	3.1	0.9
**Mesolithic-Neolithic Transition human ($\delta^{13}C$, $\delta^{15}N$, $\delta^{35}S$)**			
Sus scrofa (wild boar)	0.9	62.1	28.4
Cervus elaphus (red deer)	2.1	62.0	32.4
Delphinidae (large-sized)	0.6	37.2	15.2
Delphinidae (medium-sized)	0.4	29.4	11.7
Epinephelus spp. (grouper)	0.4	33.6	12.3

3. Radiocarbon dating

Supplementary Table 14 │ AMS radiocarbon dates on bone collagen of humans, grouper and cetaceans from Grotta dell'Uzzo pretreated at the Max Planck Institute for Evolutionary Anthropology (Leipzig)

MPI laboratory code	context	species	% collagen yield	AMS radiocarbon laboratory code	^{14}C date (BP)	calendar age cal. BP (68.2%)	calendar age cal. BP (95.4%)
S-EVA 2774	S-01	*Homo sapiens*	1.3	KIA-36038	6140±60	7160-6950	7240-6850
S-EVA 2778	W-02	*Homo sapiens*	1.3	KIA-36701	6270±35	7250-7170	7280-7020
S-EVA 8772	X-21	*Homo sapiens*	1.3	OxA-V-2365-38	6937±33	7800-7700	7840-7680
S-EVA 8013	F-09	*Homo sapiens*	3.2	OxA-V-2364-40	7006±34	7930-7790	7940-7750
S-EVA 8010	F-12	*Homo sapiens*	1.4	OxA-V-2364-43	7753±36	8460-8340	8550-8280
S-EVA 8012	F-16	*Homo sapiens*	0.8	OxA-V-2364-41	7871±37	8720-8590	8950-8550
S-EVA 8773	Y-03	*Homo sapiens*	1.0	OxA-V-2365-39	7971±37	8980-8770	9000-8650
S-EVA 8014	Uzzo X	*Homo sapiens*	0.6	MAMS-11087	8856±37	10160-9890	10160-9770
S-EVA 2777	H-mix	*Homo sapiens*	1.8	MAMS-11084	9275±36	10550-10410	10580-10290
S-EVA 2757	Uzzo 6	*Homo sapiens*	2.2	KIA-36036	9365±40	10660-10520	10700-10440
S-EVA 2285	F-02	*Epinephelus* sp.	1.9	KIA-36035	6985±40	7490-7360	7550-7290
S-EVA 2215	F-09	*Epinephelus* sp.	1.5	KIA-36033	7020±45	7530-7400	7570-7320
S-EVA 2214	F-11	*Epinephelus* sp.	1.1	KIA-36032	7175±45	7650-7510	7710-7440
S-EVA 2281	F-14	*Epinephelus* sp.	1.0	KIA-36034	7730±80	8220-8000	8320-7930
S-EVA 25630	F-12	Mysticeti	5.4	MAMS-16238	7957±25	8470-8270	8550-8170
S-EVA 25631	U-mix	Delphinidae	13.6	MAMS-16239	8083±26	8580-8380	8730-8300

The dates were performed at the Liebniz Laboratory of the Christian Albrechts Universität of Kiel (KIA), at the Klaus Tschira Laboratory of the Curt-Engelhorn-Zentrum Archaeometrie in Mannheim (MAMS) and at the Oxford Radiocarbon Accelerator Unit (OxA). Dates were calibrated with the OxCal 4.2 software[30], using the IntCal13 curve for terrestrial fauna and the Marine13 curve for marine fauna[31]. The date on the bone collagen of the human from Trench F spit 12 (S-EVA 8010) was calibrated using both curves, because this individual consumed ~40-49% marine protein (as shown by the IsoSource 1.3.1 and FRUITS mixing model). We have used the marine curve by 40±10%, allowing for a 10% uncertainty in the mixing ratio due to difficulties in estimating the trophic level effect in $\delta^{13}C$ ratios, even in the best-controlled archaeological systems. The calendar ages of the cetaceans, which are known to be migratory, and of the human who consumed their meat (S-EVA 8010), were corrected for the reservoir effect using the correction estimated by Reimer and McCormac[32] for the Mediterranean Basin ($\Delta R = 58 \pm 85$ ^{14}C yr). The reservoir correction adopted for the dates on grouper (*Epinephelus* spp.), a non-migratory fish, was that proposed by Siani et al.[33] for Sicily ($\Delta R = 71 \pm 50$ ^{14}C yr). The 8 dates from Trench F were used with those listed in Supplementary Table 15 for the Bayesian analysis in Supplementary Fig. 3.

Supplementary Table 15 │ AMS radiocarbon dates on marine shells of *Phorcus turbinatus* and conventional radiocarbon dates on wood charcoal from Trench F at Grotta dell'Uzzo

context	material dated	radiocarbon laboratory code	^{14}C date (BP)	calendar age cal. BP (1 σ)	calendar age cal. BP (2 σ)
F-07	marine shell	OxA-13808	7173±37	7640-7510	7690-7450
F-10	marine shell	OxA-13661	7410±32	7880-7720	7930-7670
F-12	marine shell	OxA-13662	7744±33	8200-8030	8290-7990
F-18	marine shell	OxA-13419	8745±55	9440-9260	9490-9130
F-22	marine shell	OxA-13441	9720±50	10640-10440	10720-10310
F-07/09	wood charcoal	P-2733	6750±70	7670-7560	7720-7480
F-13/14	wood charcoal	P-2734	7910±70	8970-8600	9000-8590
F-16/18	wood charcoal	P-2735	8330±80	9470-9250	9520-9090

The dates on the shells were performed at the Oxford Radiocarbon Accelerator Unit (OxA)[19] and those on wood charcoal at the Radiocarbon Laboratory of the University of Pennsylvania[34]. All dates have been calibrated with the OxCal 4.2 software[30] using the IntCal13 curve for charcoal and the Marine13 curve for shells[31]. The reservoir correction adopted for the shell dates was that estimated by Siani et al.[33] for Sicily (ΔR = 71±50 ^{14}C yr), given that *P. turbinatus* is a sessile intertidal gastropod. These dates listed in this table were used with those on bone collagen for the Bayesian analysis in Supplementary Fig. 3.

Supplementary Table 16 │ Bayesian model of the 'stranding episode' based on the dates of the Mesolithic-Neolithic transitional human, mysticete and delphinid

boundary / sample	^{14}C date (BP)	unmodelled calibration BP (1σ)	unmodelled calibration BP (2σ)	modelled calibration BP (1σ)	modelled calibration BP (2σ)
END Mesolithic-Neolithic transition boundary				8410-8210	8510-7770
S-EVA 8010 *Homo sapiens* OxA-V-2364-43	7753 ± 36	8460-8340	8550-8280	8440-8350	8520-8320
S-EVA 25630 Mysticeti MAMS-16238	7957 ± 25	8440-8270	8500-8170	8430-8300	8530-8220
S-EVA 25631 Delphinidae MAMS-16239	8083 ± 26	8580-8380	8730-8300	8520-8360	8630-8320
START Mesolithic-Neolithic transition boundary				8620-8380	9070- …..

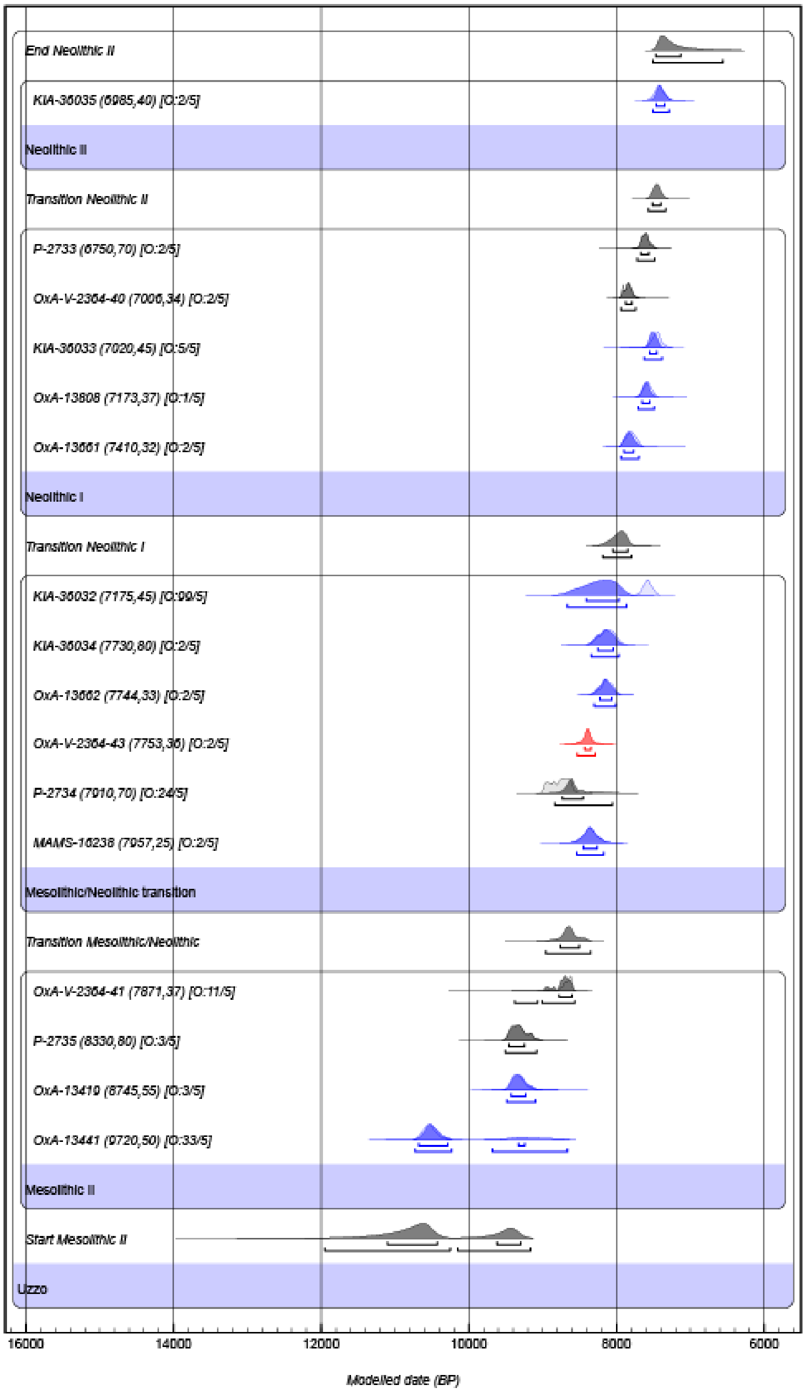

Supplementary Figure 3 │ Bayesian model of the calibrated radiocarbon dates obtained on organic remains from Trench F at Grotta dell'Uzzo. The t-type outlier model with prior probabilities set at 0.05 was used to detect problematic samples within the Bayesian model. Three samples had significant posterior probabilities indicative of outliers: OxA-13441, P-2734, KIA-36032. The dates and calibrations are in Supplementary Tables 14 and 15. Details on how they were calibrated are in the captions to these tables.

4. Location and number of cetacean strandings

Hypotheses on the likelihood that during the so-called Mesolithic-Neolithic transition hunter-gatherers had access to many individuals of the odontocete taxa recovered at Grotta dell'Uzzo can be advanced on the basis of knowledge of their behaviour and ecology. *Gl. melas*, *Gr. griseus* and *D. delphis*, for instance, are highly gregarious Delphinidae, living in organized social groups of tens, if not hundreds, of individuals[35]. This makes them susceptible to drive hunting and mass stranding, which occurs when two or more individuals strand together[36].

Odontocetes, and mainly offshore delphinids (e.g. *Gl. melas*, *Gr. griseus*, *D. delphis*), are the cetaceans that most frequently mass strand live and they do so repeatedly at similar locations or hotspots[36] (Supplementary Fig. 4). An important factor favouring this is that these marine mammals are not accustomed to shallow waters and their echolocation is impaired when inshore, particularly at times of distress and in bays that are 'acoustical dead zones'[37]. Analogies between the Gulf of Castellammare in NW Sicily and stranding hotspots worldwide, allow us to hypothesize that the cetaceans exploited by the latest hunter-gatherers of Grotta dell'Uzzo were beached at or just south of Scopello (Supplementary Fig. 5a). This locality was called *Cetaria* in antiquity, as shown in Supplementary Fig. 5b, which is part of a 19th century incision by G.R. Aloja reconstructing Sicily during Roman times and published by La Duca[38]. A tuna-fishery plant operated from this locality at least since then, exploiting the clockwise migration of bluefin tuna (*Thunnus thynnus*) around the Gulf of Castellammare during late spring and early summer[38]. At the lower sea levels of around 8,200 years ago (circa -30 m)[39], stacks at this locality (Supplementary Fig. 6), in combination with problems in echolocation linked to the likely presence of an 'acoustical dead zone'(Supplementary Fig. 4), may have constituted insurmountable obstacles for cetaceans swimming northwards parallel to the San Vito lo Capo peninsula and trying to find their way back out of the Gulf of Castellammare. In fact, the stacks at Scopello are part of a landslide that starts inland and extends seawards, one kilometer of which is now

submerged[40] and therefore were a potential obstacle even at lower sea levels. It is unlikely that cetaceans stranded closer to Grotta dell'Uzzo, because the bathymetry of the shores in its vicinity is steep and the continental platform only widens considerably south of Punta Leone, which is roughly halfway between the cave and Scopello. The area south of Scopello was within the territorial radius of the hunter-gatherers of Grotta dell'Uzzo, who likely acquired lithic raw materials just south of it at the mouth of the stream that flows into the sea at Guidaloca[41-43] and who exploited birds from coastal marshes and aquatic habitats (e.g. crakes, common moorhen, little bittern, bean goose) further south in the Gulf of Castellammare[1,2].

Statistics on animals stranded at hotspots are sparse and unsystematically published. For instance, data for Massachusetts (www.nmfs.noaa.gov/pr/health/capecod_mass_stranding_factsheet2012.pdf), but mainly relative to Cape Cod Bay (Supplementary Fig. 4b), indicate that mass strandings occur yearly (mean = 9.5 events/year) and involve on average 53.8 animals. The three cetaceans most frequently implicated were: common dolphins (*D. delphis*), Atlantic-sided dolphins (*Lagenorhynchus acutus*) and long-finned pilot whales (*Gl. melas*). Anthropogenic causes for strandings cannot be excluded outright, but this is a useful example of how live cetaceans can strand annually in high numbers within confined areas. Another example is represented by stranding data for *Gl. melas* acquired in New Zealand between 1976 and 2000 by the Department of Conservation (www.doc.govt.nz/Documents/science-and-technical/SciencePoster52.pdf). Over these 25 years, 165 events occurred, of which 83 were strandings of two or more individuals and 82 of single animals. Around half (48%) of these events concentrated at localities with gently-sloping coastal topographies (i.e. Northland, Nelson and Chatham Island regions) and at least 34% were of live animals.

At a hotspot around St Helena Bay in South Africa, 139 events occurred and a total of 203 cetaceans stranded in 1963-1981, averaging 10.7 animals per year[6]. Of 26 cetacean species from the area, 7 had

stranded in more than 10 individuals during the period of observation (including *D. delphis* and *P. macrocephalus*). Stranded cetaceans would have constituted an important resource for human groups living in the area, where strandings are seasonally predictable and often involve many individuals[3].

The data on hotspots, where offshore odontocetes strand alive in pods, give us some idea on the scale of stranding events at such localities. Even in the Mediterranean, it has been observed that when sperm whales strand live, in around a third of cases (29%) two or more individuals are involved[44]. It is, therefore, entirely feasible ecologically that, had the Gulf of Castellammare been an ephemeral hotspot, numerous live or freshly-dead odontocetes would have been available for the hunter-gatherers of NW Sicily to exploit annually. Events on this scale and with this frequency would have been necessary to explain the isotope values of the transitional hunter-gatherer from Grotta dell'Uzzo. Mysticetes, on the other hand, strand infrequently and are usually dead once beached. The isotopic data show that mysticetes may have stranded frequently enough, around the 8.2-kyr-BP event, for a terrestrial carnivore, such as *V. vulpes*, to acquire about half its dietary protein from their meat. The fact that humans consumed less mysticete meat is compatible with the lower stranding frequencies of Mysticeti and with avoidance practices linked to the dangers inherent in consuming carcasses of decomposing cetaceans.

Mass strandings are rare events in the Mediterranean[44-46], although this may be due in part to the low numbers of cetaceans now present in this sea. Strandings are more frequent in the western Mediterranean, where cetacean biodiversity and density are higher, but are not strongly clustered and there are no obvious hotspots[47]. The existence of a stranding hotspot in NW Sicily has important implications for our knowledge of cetacean distribution in the past within the Mediterranean and may imply higher population densities than at present.

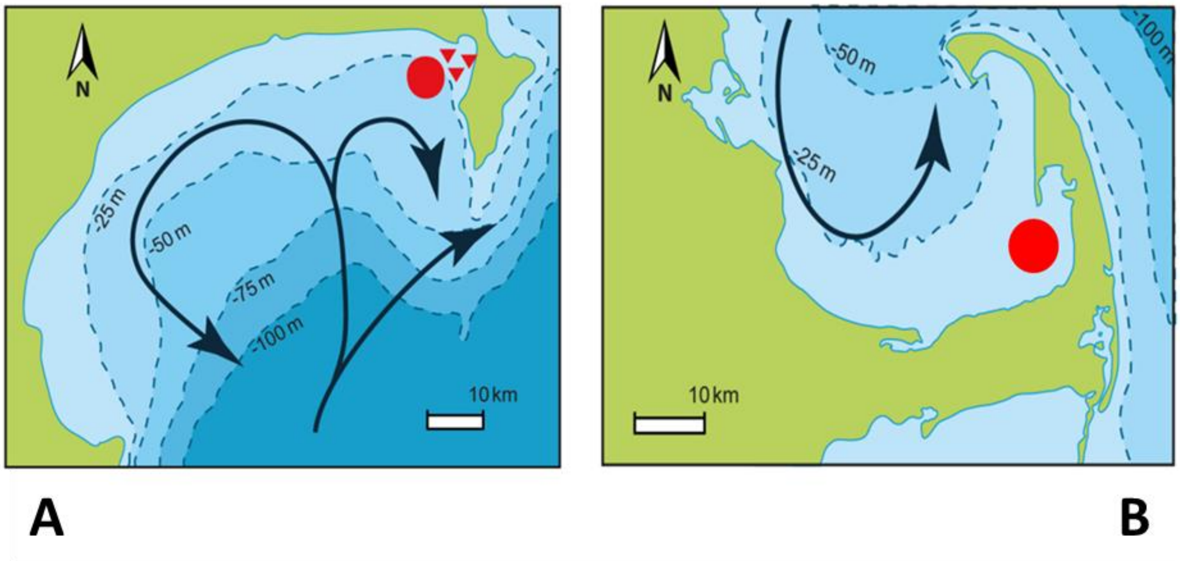

Supplementary Figure 4 │ Cetacean stranding hotspots at (A) Hawke Bay (New Zealand) and (B) Cape Cod Bay (U.S.A) and acoustical analysis of these shores. The coastal configuration of localities where mass strandings occur regularly share similar morphology, bathymetry and current movements, as Hawke and Cape Cod bays. At these shores, strandings take place mainly on the opposite side to where the currents enter the bay, as illustrated in the maps by red circles and triangles[36]. Analysis of the return rates of acoustical signals show these are 'acoustical dead zones' (red false colour in fig. 2 in Sundaram et al.[37]), as opposed to areas where echolocation is favoured by long return times (blue false colour in fig. 2 in Sundaram et al.[37]). The maps were generated using Adobe Illustrator CS.

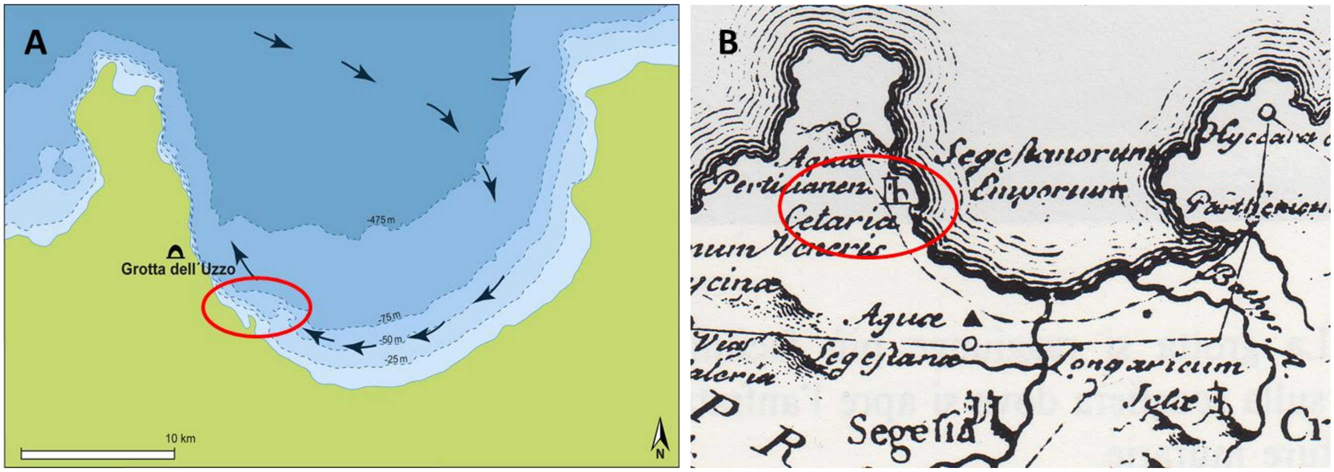

Supplementary Figure 5 │ Hypothesized location of cetacean strandings (A) and of the settlement of *Cetaria* (B). A. Gulf of Castellammare (NW Sicily) with the possible locality of the cetacean strandings corresponding to the area around the *tonnara* (tuna-fishery plant) of Scopello, which (as shown in B.) in antiquity was intriguingly called *Cetaria* (from the ancient Greek κῆτος: cetacean, sea monster or large fish). Map A has been drawn by reducing sea levels by ~30m, so that the shore is closer to that of around 8,200 years ago based on the sea level curve for NW Sicily[39]. It is worth noting that this crude reconstruction of the past shorelines shows that the area around Scopello was characterized by a protruding strip of land, coinciding with a now submerged landslide. The arrows in map A show the direction of the migration around the bay by bluefin tuna (*Thunnus thynnus*), pursuing their prey and exploiting surface currents[38]. Stranded cetaceans around 8,200 years ago may have moved similarly. Map B is part of a 19th century incision by G.R. Aloja[38] reconstructing NW Sicily during Roman times on the basis of Ptolemy's *Geographia*. Map A was generated using Adobe Illustrator CS.

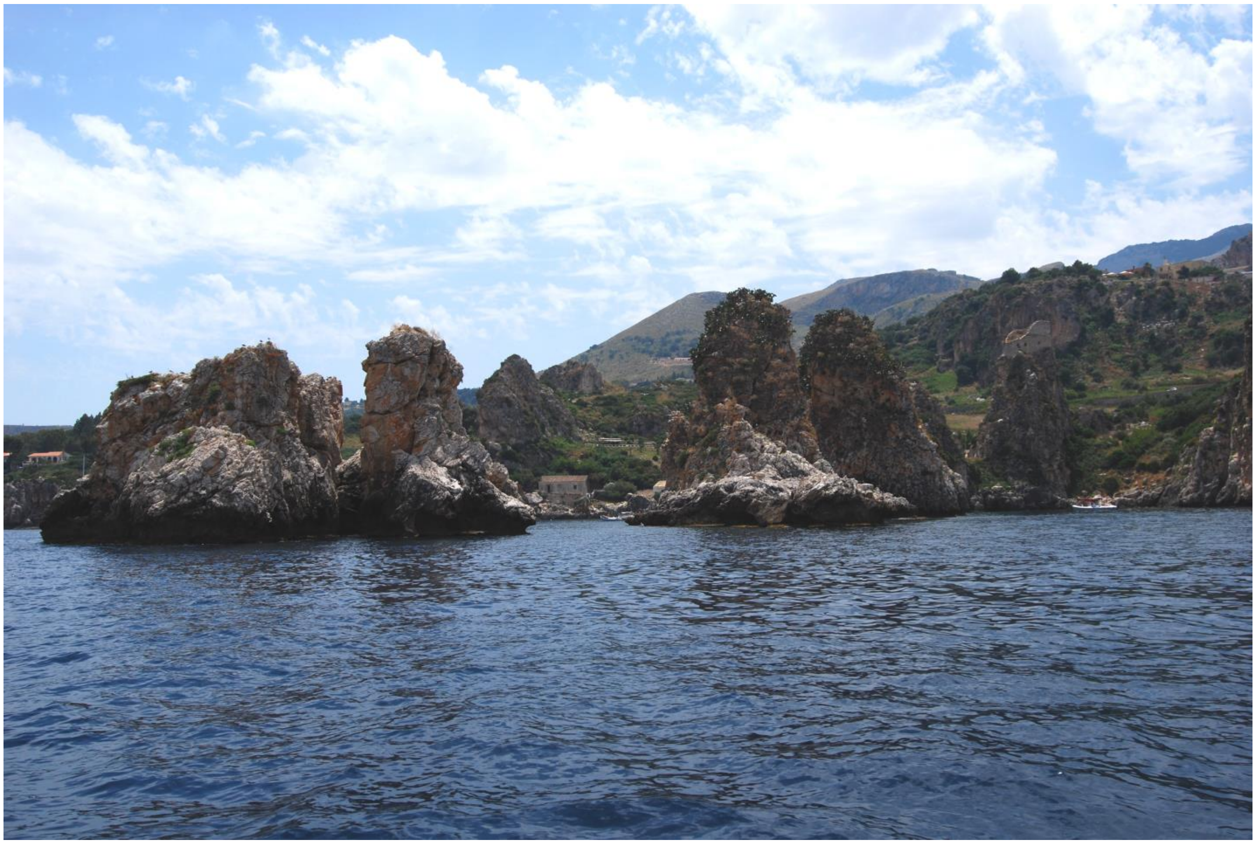

Supplementary Figure 6 | Stacks off the coast of Scopello. These are part of a landslide that starts inland and extends seawards, currently submerged up to about a kilometer from the present-day shoreline[40]. This is a view of Scopello from a boat stationing north of the stacks, which large fish and marine mammals migrating clockwise from the south of the Gulf of Castellammare have to contend with. The presence of this landslide perpendicular to the shore, as well as analogies with bays that are stranding hotspots, suggest that cetaceans may have stranded south of Scopello. The photo was taken by Marcello A. Mannino.

Supplementary Information References

1. Tagliacozzo, A. Archeozoologia della Grotta dell'Uzzo, Sicilia. Da un'economia di caccia ad un'economia di pesca ed allevamento. *Supplemento del Bullettino di Paletnologia Italiana* **84**. (Poligrafico e Zecca dello Stato, Roma, 1993).

2. Tagliacozzo, A. Economic changes between the Mesolithic and the Neolithic in the Grotta dell'Uzzo (Sicily, Italy). *Accordia Research Papers* **5**, 7-37 (1994).

3. Notarbartolo di Sciara, G. (ed) *Cetaceans of the Mediterranean and Black Seas: state of knowledge and conservation strategies* (ACCOBAMS Secretariat, Monaco, 2002).

4. Rowley-Conwy, P., Halstead, P. & Collins, P. Derivation and application of a Food Utility Index (FUI) for European wild boar (*Sus scrofa* L.). *Environ. Archaeol.* **7**, 77-87 (2002).

5. Savelle, J.M. & Friesen, T.M. An Odontocete (Cetacea) Meat Utility Index. *J. Archaeol. Sci.* **23**, 713-721 (1996).

6. Smith, A.B. & Kinahan, J. The invisible whale. *World. Archaeol.* **16**, 89-97 (1984).

7. McCartney, A.P. The nature of Thule Eskimo whale use. *Arctic* **33**, 517-541 (1980).

8. Albertini, D. & Tagliacozzo, A. in *Atti del 2° Convegno Nazionale di Archeozoologia (Asti, 1997)* (eds. Malerba, G., Cilli, C. & Giacobini, G.)(Abaco Edizioni, Forlì, 2000).

9. Mustoni, A., Pedrotti, L., Zanon, E. & Tosi, G. *Ungulati delle Alpi* (Nitida Immagine Editrice, Cles (Trento), 2002).

10. Macdonald, D.W. *The Encyclopedia of Mammals,* 3 ed. (Oxford Univ Press, Oxford, 2012).

11. Lyman, R.L. *Quantitative Paleozoology* (Cambridge Univ Press, Cambridge, 2008).

12. Casteel, R.W. Faunal assemblages and the "Wiegemethode" or Weight Method. *J. Field Archaeol.* **5**, 71-77 (1978).

13. Nehlich, O., Barrett, J.H. & Richards, M.P. Spatial variability in sulphur isotope values of archaeological and modern cod (*Gadus morhua*). *Rapid. Commun. Mass Sp.* **27**, 2255-2262 (2013).

14. Vika, E. & Theodoropoulou, T. Re-investigating fish consumption in Greek antiquity: results from $\delta^{13}C$ and $\delta^{15}N$ analysis from fish bone collagen. *J. Archaeol. Sci.* **39**, 1618-1627 (2012).

15. Pinela, A.M., Borrell, A., Cardona, L. & Aguilar, A. Stable isotope analysis reveals habitat partitioning among marine mammals off the NW African coast and unique trophic niches for two globally threatened species. *Mar. Ecol. Prog. Ser.* **416**, 295-306 (2010).

16. Pantoja, S., Repeta, D.J., Sachs, J.P. & Sigman, D.M. Stable isotope constraints on the nitrogen cycle of the Mediterranean Sea water column. *Deep-Sea Res. Pt. I* **49**, 1609-1621 (2002).

17. Francalacci, P. Comparison of archaeological trace element and stable isotope data from two Italian coastal sites. *Rivista di Antropologia* **56**, 239-250 (1988).

18. Mannino, M.A., *et al*. Origin and diet of the prehistoric hunter-gatherers on the Mediterranean Island of Favignana (Ègadi Islands, Sicily). *PLoS ONE* **7** (11), e49802 (2012).

19. Mannino, M.A., *et al*. Marine resources in the Mesolithic and Neolithic at the Grotta dell'Uzzo (Sicily): evidence from isotope analyses of marine shells. *Archaeometry* **49**, 117-133 (2007).

20. Lelli, R., *et al*. Examining dietary variability of the earliest farmers of south-eastern Italy. *Am. J. Phys. Anthropol.* **149**, 380-390 (2012).

21. Borgognini Tarli, S.M., Canci, A. & Piperno, M. Dati archeologici e antropologici sulle sepolture mesolitiche della Grotta dell'Uzzo (Trapani). *Bullettino di Paletnologia Italiana* **84**, 85-179 (1993).

22. Di Salvo, R., Mannino, M.A., Schimmenti, V., Sineo, L. & Thomas, K.D. Nuovi dati sulle sepolture della Grotta dell'Uzzo. *Atti della XLI Riunione Scientifica, Istituto Italiano di Preistoria e Protostoria*, 461-470 (2012).

23. Gannier, A., Drouot, V. & Goold, J.C. Distribution and relative abundance of sperm whales in the Mediterranean Sea. *Mar. Ecol. Prog. Ser.* **243**, 281-293 (2002).

24. Bentaleb, I., *et al*. Foraging ecology of Mediterranean fin whales in a changing environment elucidated by satellite tracking and baleen plate stable isotopes. *Mar. Ecol. Progr. Ser.* **438**, 285-302 (2011).

25. Phillips, D.L. & Gregg, J.W. Source partitioning using stable isotopes: coping with too many sources. *Oecologia* **136**, 261-269 (2003).

26. Fernandes, R., Millard, A.R., Brabec, M., Nadeau, M.-J. & Grootes, P. Food Reconstruction Using Isotopic Transferred Signals (FRUITS): a Bayesian model for diet reconstruction. *PLoS ONE* **9** (2), e87436 (2014).

27. Britton, K., *et al*. Maritime adaptations and dietary variation in prehistoric western Alaska: stable isotope analysis of permafrost-preserved human hair. *Am. J. Phys. Anthr.* **151**, 448-461 (2013).

28. Lee-Thorp, J.A. On isotopes and old bones. *Archaeometry* **50**, 925-950 (2008).

29. Bocherens, H. & Drucker, D. Trophic level isotopic enrichment of carbon and nitrogen in bone collagen: case studies from recent and ancient terrestrial ecosystems. *Int. J. Osteoarch.* **13**, 46-53 (2003).

30. Bronk Ramsey, C. & Lee, S. Recent and planned developments of the program OxCal. *Radiocarbon* **55**, 720-730 (2013).

31. Reimer, P.J., *et al*. IntCal13 and Marine13 radiocarbon age calibration curves 0-50,000 years cal BP. *Radiocarbon* **55**, 1869-1887 (2013).

32. Reimer, P.J. & McCormac, F.G. Marine radiocarbon reservoir corrections for the Mediterranean and Aegean seas. *Radiocarbon* **44**, 159-166 (2002).

33. Siani, G., *et al*. Radiocarbon reservoir ages in the Mediterranean Sea and Black Sea. *Radiocarbon* **42**, 271-280 (2000).

34. Meulengracht, A., McGovern, P. & Lawn, B. University of Pennsylvania radiocarbon dates XXI. *Radiocarbon* **23**, 227-240 (1981).

35. Wade, P.R., Reeves, R.R. & Mesnick, S.L. Social and behavioural factors in Cetacean responses to overexploitation: are odontocetes less “resilient” than Mysticetes? *J. Mar. Biol.* doi:10.1155/2012/567276 (2012).

36. Brabyn, M.W. & McLean, I.G. Oceanography and coastal topography of herd-stranding sites for whales in New Zealand. *J. Mammal.* **73**, 469-476 (1992).

37. Sundaram, B., Poje, A.C., Veit, R.R. & Nganguia, H. Acoustical dead zones and the spatial aggregation of whale strandings. *J. Theoret. Biol.* 238, 764-770 (2006).

38. La Duca, R. *La Tonnara di Scopello* (Edizioni Grifo, Palermo, 1988).

39. Lambeck, K., Antonioli, F., Purcell, A. & Silenzi, S. Sea level change along the Italian coast for the past 10,000yr. *Quat. Sci. Rev.* **23**, 1567-1598 (2004).

40. Agate, M., *et al*. I fondali marini della Penisola di San Vito (Sicilia nord-occidentale). Atti del 4° Convegno di Speleologia della Sicilia. *Speleologia Iblea* **10**, 83-95 (2004).

41. Collina, C. Gestione della materia prima e sistemi tecnici alla Grotta dell’Uzzo. Risultati dell’analisi petrografica e tecnologica dell’industria litica dei livelli neolitici. *Atti della XXXIX Riunione Scientifica, Istituto Italiano di Preistoria e Protostoria*, 472-476 (2006).

42. Collina, C. *Evolution des industries lithiques du Néolithique ancien en Italie du sud* (Thèse de Doctorat, Université de Provence and Università degli Studi di Roma ‘La Sapienza’, 2009).

43. Collina, C. Sistemi tecnici e chaînes opératoires alla grotta dell’Uzzo (TP). Analisi tecnologica delle industrie litiche dai livelli mesolitici e neolitici. *Atti della XLI Riunione Scientifica, Istituto Italiano di Preistoria e Protostoria*, 447-459 (2012).

44. Bearzi, G., *et al*. Overview of sperm whale *Physeter macrocephalus* mortality events in the Adriatic Sea, 1555-2009. *Mammal Rev.* **41**, 276-293 (2011).

45. Bearzi, G., Reeves, R.R., Remonato, E., Pierantonio, N. & Airoldi, S. Risso's dolphin *Grampus griseus* in the Mediterranean Sea. *Mammal. Biol.* **76**, 385-400 (2011).

46. Mazzariol, S., *et al*. Sometimes sperm whales (*Physeter macrocephalus*) cannot find their way back to the high seas: a multidisciplinary study on a mass stranding. *PLoS ONE* **6** (5), e19417 (2011).

47. Rojo-Nieto, E., *et al*. Strandings of cetaceans and sea turtles in the Alboran Sea and Strait of Gibraltar: a long-term glimpse at the north coast (Spain) and the south coast (Morocco). *Anim. Biodiver. Conser.* **34**, 151-163 (2011).
